# Molecular insights into the enzymatic diversity of flavin‐trafficking protein (Ftp; formerly ApbE) in flavoprotein biogenesis in the bacterial periplasm

**DOI:** 10.1002/mbo3.306

**Published:** 2015-12-02

**Authors:** Ranjit K. Deka, Chad A. Brautigam, Wei Z. Liu, Diana R. Tomchick, Michael V. Norgard

**Affiliations:** ^1^ Department of MicrobiologyThe University of Texas Southwestern Medical CenterDallasTexas75390; ^2^ Department of BiophysicsThe University of Texas Southwestern Medical CenterDallasTexas75390

**Keywords:** FAD pyrophosphatase, flavoprotein, FMN transferase, lipoprotein, posttranslational modification, redox protein

## Abstract

We recently reported a flavin‐trafficking protein (Ftp) in the syphilis spirochete *Treponema pallidum* (Ftp_Tp) as the first bacterial metal‐dependent FAD pyrophosphatase that hydrolyzes FAD into AMP and FMN in the periplasm. Orthologs of Ftp_Tp in other bacteria (formerly ApbE) appear to lack this hydrolytic activity; rather, they flavinylate the redox subunit, NqrC, via their metal‐dependent FMN transferase activity. However, nothing has been known about the nature or mechanism of metal‐dependent Ftp catalysis in either Nqr‐ or Rnf‐redox‐containing bacteria. In the current study, we identified a bimetal center in the crystal structure of *Escherichia coli* Ftp (Ftp_Ec) and show via mutagenesis that a single amino acid substitution converts it from an FAD‐binding protein to a Mg^2+^‐dependent FAD pyrophosphatase (Ftp_Tp‐like). Furthermore, in the presence of protein substrates, both types of Ftps are capable of flavinylating periplasmic redox‐carrying proteins (e.g., RnfG_Ec) via the metal‐dependent covalent attachment of FMN. A high‐resolution structure of the Ftp‐mediated flavinylated protein of *Shewanella oneidensis* NqrC identified an essential lysine in phosphoester‐threonyl‐FMN bond formation in the posttranslationally modified flavoproteins. Together, these discoveries broaden our understanding of the physiological capabilities of the bacterial periplasm, and they also clarify a possible mechanism by which flavoproteins are generated.

## Introduction

Flavin is an essential cofactor required for metabolic processes within all living organisms (Fischer and Bacher 2005; Macheroux et al. 2011). Because of its ability to exist in either fully oxidized, one‐electron reduced, or two‐electron reduced states, protein‐bound flavin can serve as a redox carrier for a wide variety of enzymatic reactions. Despite this metabolic versatility, posttranslational flavinylation as well as flavoprotein biogenesis have been under‐studied, and they had been thought not to occur in the bacterial periplasm. This latter assumption may have been fueled by a previous annotation of a large family of bacterial periplasmic proteins originally designated as “alternative pyrimidine biosynthesis enzymes” (ApbE superfamily) that were thought to be involved in thiamine biosynthesis (Beck and Downs 1998, 1999). In addition, the bacterial periplasm has rarely been investigated as a metabolically active subcellular flavin‐based redox compartment, and the ApbE family of proteins had not been characterized functionally or biochemically until others and we established their roles in periplasmic flavin homeostasis and flavoprotein biogenesis (Bertsova et al. 2013; Deka et al. 2013a). From recent structure‐function analyses, we identified in *Treponema pallidum*, the noncultivable syphilis spirochete, an ApbE ortholog (TP0796) which we have renamed flavin‐trafficking protein (Ftp_Tp) and that functions as a Mg^2+^‐dependent FAD hydrolase (Deka et al. 2013a). Newly emerging evidence now supports the notion that Ftp‐like proteins play an important role in flavoprotein biogenesis in the bacterial periplasm (Bertsova et al. 2013; Deka et al. 2013a, 2015; Steuber et al. 2014). Recently, two flavin‐ and quinone‐based redox‐driven Na^+^‐pumps (Nqr, Na^+^‐translocating NADH:quinone oxidoreductase and Rnf, *Rhodobacter* nitrogen fixation) have been discovered that are believed to be of central importance to the bioenergetics of many pathogenic bacteria, and often they are the only ion‐motive electron‐transport chain in these organisms (Biegel et al. 2011). To possess the appropriate electron carrying functions, some subunits (RnfG and NqrC) of the redox complexes require protein‐mediated posttranslational flavinylation in the periplasm, providing a strategic link between those functions and redox reactions that typically occur within the bacterial cytoplasm (Steuber et al. 2014). We initially coined the term “flavin‐trafficking protein” (Ftp) to reflect its proposed strategic role in both flavin homeostasis and flavoprotein biogenesis (Deka et al. 2013a, 2015); this has prompted further investigations aimed at delineating Ftp's overall role in flavoprotein biogenesis in the periplasm.

There are two types of periplasmic Ftp‐like proteins (Boyd et al. 2011; Bertsova et al. 2013; Deka et al. 2013a). Whereas Ftp from *T. pallidum* (Ftp_Tp) displays Mg^2+^‐dependent FAD pyrophosphatase (EC 3.6.1.18) activity that hydrolyzes FAD into AMP and FMN (Deka et al. 2013a, 2015), orthologs of Ftp_Tp from other bacteria appear to lack the hydrolytic activity. Rather, they bind FAD and flavinylate (FMN transferase, EC 2.7.1.180) a subunit (NqrC) of a cytoplasmic membrane redox system (Nqr); this occurs despite significant structural similarity (approximately 25–30% sequence identity) to Ftp_Tp. However, prior studies have focused largely on the flavoprotein products of the reactions associated with the Nqr‐redox system (Borshchevskiy et al. 2015), rather than the overall mechanism of FMN transfer between the donor‐acceptor protein catalyzed reactions.

To investigate the functional diversity and the molecular mechanism(s) of Ftp‐mediated protein flavinylation, this study employed both biochemical analyses and structure‐based mutagenesis on various Ftp orthologs (formerly ApbEs). We found that a single amino acid change in *Escherichia coli* Ftp (Ftp_Ec) converts it from an FAD‐binding protein to a Mg^2+^‐dependent FAD pyrophosphatase without altering the Mg^2+^‐dependent FMN transferase activity to the RnfG_Ec subunit of the Rnf_Ec‐redox system. This finding represents the first structural demonstration of a bi‐metal catalytic center of an Ftp that mediates metal‐dependent protein flavinylation in Rnf‐redox‐containing bacteria. Furthermore, a high‐resolution structure of an Ftp‐generated NqrC flavoprotein of *Shewanella oneidensis* (NqrC_So) not only provided atomic detail of phosphoester‐threonyl‐FMN bond formation in a posttranslationally modified flavoprotein, but also has revealed new mechanistic insights into the interaction and association of donor‐acceptor proteins during the FMN‐transfer reaction. Finally, inasmuch as this type of periplasmic protein flavinylation does not occur in mammals, this new mechanistic understanding of bacterial flavoprotein biogenesis may give rise to new strategies (new potential targets) for nontraditional antimicrobial drug design.

## Materials and Methods

### Reagents

Unless otherwise noted, chemicals were either purchased from Sigma–Aldrich or Hampton Research. All oligonucleotide primers employed in this study were synthesized at Integrated DNA technologies (Coralville, IA).

### Protein preparation

Recombinant and ligand‐free Ftp_Ec protein preparations were as previously described (Deka et al. 2013a). The Ftp orthologues (a.k.a. ApbE) from *Alcanivorax borkumensis* (Abo_1038, referred to as Ftp_Ab), *Enterococcus faecalis* (EF3255, referred to as Ftp_Ef), *Haemophilus ducreyi* (HD0386, referred to as Ftp_Hd), *Listeria monocytogenes* (LMOF2365_2609, referred to as Ftp_Lm), *Shewanella oneidensis* (SO_1109, referred to as Ftp_So), and *Treponema denticola* (TDE2614, referred to as Ftp_Td) were employed in this study. In addition, the putative flavoproteins RnfG from *E. coli* (BL21_01591, referred to as RnfG_Ec), and NqrC from *S. oneidensis* (SO_0904, referred to as NqrC_So) were used. Recombinant plasmids for *ftp_Ab* (residues encoding 21 ‐ 338), *ftp_Ef* (residues encoding 21 ‐ 356), *ftp_Hd* (residues encoding 17 ‐ 343), *ftp_Lm* (residues encoding 22 ‐ 361), *ftp_So* (residues encoding 32 ‐ 348), *ftp_Td* (residues encoding 28 ‐ 372), *rnfG_Ec* (residues encoding 26 ‐ 207) and *nqrC_So* (residues encoding 32 ‐ 265) were generated using the polymerase incomplete primer extension (PIPE) cloning method (Klock et al. 2008). Genes encoding truncated versions of the proteins (without their predicted N‐terminal transmembrane helices in the cases of NqrC homologs, or signal peptides including the N‐terminal acylated Cys‐residue in the cases of Ftp homologs) from their respective genomic DNA (obtained from ATCC) were amplified by PCR using *pfu*Turbo DNA polymerase (Agilent Technologies, Santa Clara, CA, USA) and primers encoding the predicted 5′ and 3′ termini of the genes (PIPE‐Inserts). The expression vector, pSpeedET (DNASU, AZ), which encodes an N‐terminal TEV‐protease cleavable expression and purification hexa‐histidine tag (MGSDKIHHHHHHENLYFQG), were PCR amplified with PIPE‐vector primers. PIPE‐inserts for respective gene insert and PIPE‐vector were individually mixed to anneal the amplified DNA fragments together. *E. coli* HK100 competent cells were transformed with the mixtures (PIPE‐vector and insert) and selected for kanamycin resistance on LB agar plates. Cloning junctions/fragments were verified by DNA sequencing. Protein expression was performed in LB media with l‐arabinose as inducers. The procedures for expression and purification of the recombinant proteins were essentially as previously described (Deka et al. 2013a, 2013b).

For the production of native and selenomethionine labeled proteins, NqrC_So was recloned into a pProEx HTb vector (Invitrogen, Grand Island, NY, USA), and proteins were overproduced as described previously (Deka et al. 2002, 2012). Purified apo proteins were then flavinylated in vitro by mixing with purified Ftp_So in the presence of FAD and MgCl_2_ as described below (Flavinylation Assay). The hexa‐histidine tag of the flavinylated NqrC_So was cleaved off with TEV protease (Invitrogen) and the flavinylated protein was further purified by anion exchange on a Mono Q HR 5/5 column (GE Life Sciences, Pittsburgh, PA, USA) followed by gel filtration chromatography (Deka et al. 2013a, 2013b).

### Site‐directed mutagenesis

Mutations were introduced into the plasmids carrying wild‐type sequences using a QuikChange site‐directed mutagenesis kit (Agilent Technologies). All protein variants were confirmed by DNA sequencing. Protein variants were expressed and purified as described for the wild‐type proteins (Deka et al. 2013a).

### Protein concentration determination and UV‐visible spectroscopy

Protein concentrations were determined in buffer A (20 mmol L^−1^ Hepes, pH 7.5, 0.1 mol L^−1^ NaCl, 2 mmol L^−1^ *β*‐octylglucoside) from their deduced extinction coefficients using the ProtParam utility of ExPASy (Gasteiger et al. 2005). UV–visible absorption spectra of yellow proteins in buffer A were recorded over the scan range 300–600 nm using a NanoDrop 2000C (Fisher Scientific, Grand Island, NY, USA).

### FAD pyrophosphatase (EC 3.6.1.18) assay

FAD pyrophosphatase activity was assayed by measuring the production of FMN formation, as described previously (Deka et al. 2013a). Briefly, the standard 200 *μ*L reaction mixture contained 1 *μ*mol L^−1^ enzyme/protein, 5 mmol L^−1^ MgCl_2_, and 10 *μ*mol L^−1^ FAD in buffer A, and was allowed to incubate at 37°C for 20 min. Inhibition reactions were preincubated either with 5 mmol L^−1^ EDTA or with 1 mmol L^−1^ AMP/ADP prior to assaying for remaining FAD hydrolytic activities (Deka et al. 2013a).

Burst kinetics were performed at room temperature (~22°C) as described by Deka et al. (2013a).

### Flavinylation (FMN transferase, EC 2.7.1.180) assay

Purified proteins in buffer A were incubated with the indicated concentrations of exogenous FAD and MgCl_2_ in a 100 *μ*L reaction volume for 1 h at 30°C. Approximately 100 *μ*mol L^−1^ of either RnfG_Ec or NqrC_So and their variants were incubated in buffer A containing ~20 *μ*mol L^−1^ of their respective Ftp_Ec/Ftp_So, 5 mmol L^−1^ MgCl_2_ and 1 mmol L^−1^ FAD. Reactions were stopped by adding equal volume of 2X‐SDS‐PAGE sample buffer and boiled for 5 min. An approximately 20 *μ*L sample of boiled reactions were separated on a 12.5% SDS PAGE and visualized by UV‐illumination with a Gel Logic 200 Imaging System (Kodak) before Coomassie Blue staining. Upon UV illumination, the flavinylated protein was visible due to the fluorescence of the covalently bound flavin. Sometimes, boiled reaction mixtures were frozen until use.

### Crystallization and data collection

The crystallization and data collection of Ftp_Ec native and protein variant crystals were obtained using the hanging‐drop vapor diffusion method at 20°C by equilibration of drops versus a reservoir solution composed of 0.2 mol L^−1^ NH_4_NO_3_ and 20% (w/v) PEG 3350. Drops contained 4 *μ*L protein (~20 mg mL^−1^) in 20 mmol L^−1^ Tris, pH 7.5, 20 mmol L^−1^ NaCl and were mixed with 4 *μ*L of reservoir solution; crystals of the ternary complex of the Ftp_Ec protein variant (Y60N) with ADP were obtained from drops that also contained 1 *μ*L of 50 mmol L^−1^ MgCl_2_ and 1 *μ*L of 50 mmol L^−1^ ADP.

The crystals of native NqrC_So were obtained using the hanging‐drop vapor diffusion method at 20°C by mixing 4 *μ*L protein (~20 mg mL^−1^) in 20 mmol L^−1^ Tris, pH 7.5, 20 mmol L^−1^ NaCl with 4 *μ*L reservoir solution composed of 25% (w/v) PEG 1500, 0.1 mol L^−1^ MIB (malonate, imidazole and boric acid) buffer pH 5.0. The crystals typically grew as flat plates and exhibit the symmetry of space group P2_1_ with four copies of NqrC_So in the asymmetric unit, and were cryoprotected by briefly dipping into the reservoir solution, then flash cooling in liquid nitrogen. Crystals of selenomethionyl‐derivatized NqrC_So were obtained by a similar method with a reservoir solution of 25% (w/v) PEG 1500, 0.1 mol L^−1^ bicine pH 9.0, 0.1 mol L^−1^ NaCl, and were cryoprotected by gradual transfer in steps of 5% ethylene glycol to a final solution of 20% (v/v) ethylene glycol, 27% (w/v) PEG 1500, 0.1 mol L^−1^ bicine pH 9.0, 0.1 mol L^−1^ NaCl. These crystals typically grew as rods and exhibit the symmetry of space group P2_1_2_1_2_1_, with six copies of NqrC_So in the asymmetric unit.

Synchrotron X‐ray diffraction data were collected at Sector 19 (Structural Biology Center) of the Advanced Photon source and were indexed, integrated and scaled using the HKL‐3000 program package (Minor et al. 2006). Data collection statistics are provided in Table 1.

**Table 1:** Data collection, phasing and refinement statistics for *E. coli* Ftp and *S. oneidensis* NqrC structures

Crystal	*E. coli* Ftp	*E. coli* Ftp E169K	*E. coli* Ftp Y60N + ADP	*S.oneidensis* NqrC, native	*S. oneidensis* NqrC, Se^1^ peak
Data collection					
Wavelength (Å)	0.97918	0.97718	0.97918	0.97918	0.97934
Space group	P1	P1	P2_1_2_1_2_1_	P2_1_	P2_1_2_1_2_1_
Cell constants a, b, c, *α*,* β*,* γ*	56.20, 70.65, 85.96, 75.70, 72.45, 69.28	57.22, 70.65, 86.28, 75.72, 71.91, 69.72	54.58, 56.98, 224.77, 90.0, 90.0, 90.0	70.91, 93.48, 71.14, 90.0, 100.24, 90.0	43.78, 132.05, 251.40, 90.0, 90.0, 90.0
Resolution range (Å)	31.5–1.88 (1.91–1.88)	33.8–1.75 (1.78–1.75)	29.6–1.85 (1.88–1.85)	33.7–1.76 (1.79–1.76)	37.2–2.82 (2.87–2.82)
Unique reflections	91,038 (4488)	116,958 (5748)	57,017 (1856)	89,381 (4319)	36,559 (1546)
Multiplicity	2.1 (2.1)	3.7 (3.6)	4.1 (3.4)	4.1 (3.4)	7.7 (5.4)
Data completeness (%)	96.9 (94.5)	97.3 (96.0)	93.6 (60.7)	99.0 (96.3)	99.2 (86.1)
*R*_merge_ (%)^2^	6.4 (58.1)	8.7 (85.5)	3.9 (60.6)	6.5 (38.4)	7.4 (30.9)
*I*/*σ*(*I*)	13.7 (1.8)	20.7 (1.8)	29.6 (1.34)	15.9 (2.56)	24.8 (4.64)
Wilson *B‐*value (Å^2^)	20.5	18.6	18.8	59.2	47.1
Phase determination					
Anomalous scatterers					Selenium, 36 out of 36 possible sites
Figure of merit (42.7–2.82 Å)					0.40

Crystal	*E. coli* ApbE	*E. coli* ApbE E169K	*E. coli* Y60N + ADP	*S. oneidensis* NqrC, native
Refinement statistics				
Resolution range (Å)	31.5–1.88 (1.90–1.88)	33.8–1.75 (1.77–1.75)	28.3–1.90 (1.94–1.90)	37.1–1.76 (1.80–1.76)
No. of reflections *R*_work_/*R*_free_	90,849/4543 (2561/148)	116,560/5873 (3192/188)	49,844/2495 (1110/56)	88,913/2032 (5722/132)
Data completeness (%)	96.4 (85.0)	96.6 (84.0)	88.4 (38.0)	98.3 (89.0)
Twin law/twin fraction	NA	NA	NA	l,‐k,h/0.380
Atoms (non‐H protein/solvent/metals/nucleotide or cofactor)	9549/832/6/0	9458/695/7/0	4512/424/4/54	6955/651/1/120
*R*_work_ (%)	16.4 (29.5)	17.2 (24.4)	18.0 (22.6)	16.6 (23.5)
*R*_free_ (%)	20.0 (39.7)	19.8 (30.0)	21.4 (24.9)	20.2 (24.8)
R.m.s.d. bond length (Å)	0.007	0.006	0.017	0.004
R.m.s.d. bond angle (°)	0.96	0.87	0.99	0.65
Mean B‐value (Å^2^) (protein/solvent/metals/nucleotide or cofactor)	30.6/33.3/26.4/NA	30.5/31.5/29.0/NA	29.7/36.3/31.1/37.2	21.2/22.5/16.5/25.2
Ramachandran plot (%) (favored/additional/disallowed)^2^	98.2/1.6/0.2	98.0/1.9/0.1	97.4/2.4/0.2	97.5/2.5/0.0
Missing residues	A: 1–11, 216**–**221, 245–246, 329–341B: 1–12, 216–223, 330–341C: 1–12, 116–118, 217–221, 330–341D: 1–12, 216–222, 243–247, 330–341	A: 1–11, 120–121, 214–222, 245–246, 329–341B: 1–12, 216–223, 330–341C: 1–12, 116–119, 217–222, 330–341D: 1–12, 216–223, 242–247, 330–341	A: 1–12, 115–121, 213–223, 242–250, 330–341B: 1–12, 116–120, 215–223, 242–251, 330–341	A: −27 to −1, 255 to 264B: −27 to −4, 254 to 264C: −27 to 0, 33 to 37, 255 to 264D: −27 to 0, 33 to 36, 256 to 264

Data for the outermost shell are given in parentheses.

^1^Bijvoet‐pairs were kept separate for data processing.

^2^ *R*_merge_ = 100 Σ_*h*_Σ_*i*_|*I*_*h*,*i*_—* *〈*I*_*h*_〉*|/*Σ_*h*_Σ_*i*_ 〈*I*_*h*,*i*_〉, where the outer sum (*h*) is over the unique reflections and the inner sum (*i*) is over the set of independent observations of each unique reflection.

### Phase determination and structure refinement

Phases for the Ftp_Ec_E169K_ protein variant structure were obtained via isomorphous replacement using the apo structure (Deka et al. 2013a) (PDB ID: 2O18). Phases for the Ftp_Ec protein variant (Y60N) complexed with ADP were obtained by the molecular replacement method in the program *Phaser* (McCoy et al. 2007) using the Ftp_Ec apo structure as a search model. Phases for NqrC_So were obtained from a single‐wavelength anomalous dispersion experiment using selenomethionyl‐labeled protein with data to a *d*_min_ of 2.82 Å. Thirty‐six selenium sites were located using the program *SHELXD* (Schneider and Sheldrick 2002), and phase refinement and density modification with sixfold noncrystallographic symmetry averaging was performed in the program suite *Phenix* (Adams et al. 2010), resulting in an overall figure of merit of 0.40 for data between 42.7 and 2.82 Å. An initial model containing 84% of all NqrC_So residues was automatically generated via the autobuilding routine of *Phenix*, and this model was used to phase the native NqrC_So structure via the molecular replacement method in the program *Phaser*. Automatic model rebuilding performed in the program *ARP/wARP* (Langer et al. 2008) yielded a model containing 83% of all NqrC_So residues in the four independent monomers of the asymmetric unit.

Manual model rebuilding was performed in the program *Coot* (Emsley et al. 2010) and refinement was performed in the program *Phenix* (Adams et al. 2010). Native NqrC_So data display reflection conditions hkl: h + l = 2*n* that are systematically weak, indicating pseudo B‐centering. Inspection of the data statistics in the *Xtriage* module of the program *Phenix* indicated that the data is pseudo‐merohedrally twinned, with a twin law of *l*, ‐*k*,* h*. Refinement of the native NqrC_So model with this twin law resulted in a decrease of *R*_free_ by 5% and a twin fraction of 0.38. Model refinement statistics are provided in Table 1.

### Protein data bank accession codes

The coordinates and structure factors for apo Ftp_Ec (4XGV), apo Ftp_Ec (E169K) protein variant (4XGW), Ftp_Ec (Y60N) protein variant‐ADP complex (4XGX) and Nqrc_So (4XHF) have been deposited in the Protein Data Bank.

## Results and Discussion

### Characterization of the recombinant proteins: two classes of Ftps

**Figure 1:**
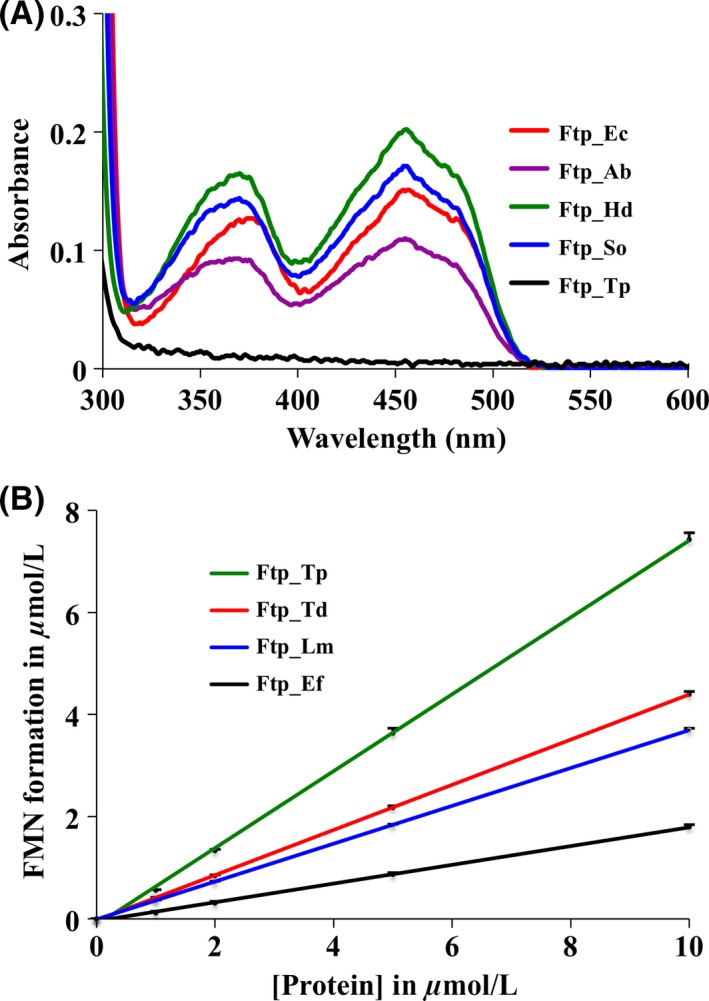
Characterization of recombinant Ftps. (A) UV–visible absorption spectra of recombinant Ftps (~16 *μ*mol L^−1^ Ftp_Ec, ~29 *μ*mol L^−1^ Ftp_Ab, ~20 *μ*mol L^−1^ Ftp_So, ~75 *μ*mol L^−1^ Ftp_Hd and ~18 *μ*mol L^−1^ Ftp_Tp), as isolated. The UV–visible absorption spectrum of Ftp_Tp is shown for comparison (*black line*). (B) FAD turnover by recombinant Ftps were measured as described previously (Deka et al. 2013a). Points are plotted as the mean of two samples after 20 min incubation at room temperature. The FAD turnover by the wild‐type TP0796 (Ftp_Tp) was published previously (Deka et al. 2013a). Data are presented as mean ± standard error (*n *=* *2). Standard error of the data points were <0.13 *μ*mol L^−1^.

All recombinant Ftps were soluble and purified as described previously (Deka et al. 2013a). *Alcanivorax borkumensis* Ftp (Ftp_Ab), Ftp_Ec, *Haemophilus ducreyi* Ftp (Ftp_Hd) and *Shewanella oneidensis* Ftp (Ftp_So) purified as yellow, flavin‐bound forms (holoproteins). As shown in Figure 1A, yellow Ftps exhibited UV‐visible absorption maxima at ~370 and ~450 nm (with a pronounced shoulder at ~480 nm), indicative of bound flavin. The observed nonstoichiometric FAD‐binding to recombinant proteins was consistent with the previous report of FAD‐binding protein of *Salmonella enterica* (Boyd et al. 2011). *Enterococcus faecalis* Ftp (Ftp_Ef), *Listeria monocytogenes* Ftp (Ftp_Lm), *Treponema denticola* Ftp (Ftp_Td), and Ftp_Tp contained no flavin (apoproteins); these nonyellow Ftps were assayed for metal‐dependent FAD pyrophosphatase activity (Fig. 1B). Similar to Ftp_Tp (Deka et al. 2013a), all nonyellow Ftps showed single‐turnover kinetics of FAD pyrophosphatase activity and were also inhibited either by AMP/ADP or EDTA (data not shown). These analyses confirmed our earlier results suggesting that there might be two classes of Ftps, one associated with FAD‐binding (yellow proteins) and the other with FAD hydrolysis (nonyellow proteins; Deka et al. 2013a). Recombinant RnfG_Ec and NqrC_So were purified as unflavinylated proteins. CD‐spectroscopy and/or size‐exclusion chromatography results on all proteins were consistent with the supposition that all proteins studied were globular and likely adopted their native folds.

### Conversion of an *E. coli* FAD‐binding Ftp (Ftp_Ec) into a Mg^2+^‐dependent FAD pyrophosphatase

**Figure 2:**
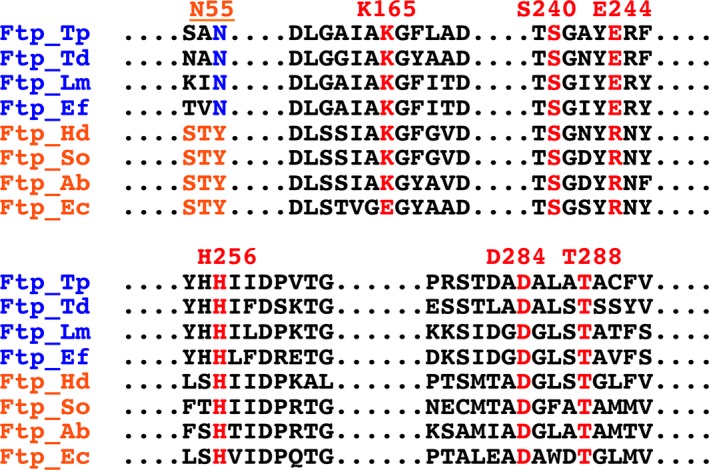
Primary sequence alignment of the Ftp enzyme active center based on the Ftp_Tp structure. The numbering is based on Ftp_Tp, and the active‐site residues are highlighted in colors. Indicated in blue lettering are the names of the Mg^2+^‐dependent FAD pyrophosphatase Ftps, and in red lettering are the names of the FAD binding Ftps. Listed above the sequence in red letters are the residues involved in either metal or FAD binding in the Ftps; underlined is the critical residue that contacts the isoalloxazine ring of FAD, which is a tyrosine residue in the FAD‐binding Ftps.

A sequence alignment of residues in the active site of Ftps employed in this study revealed two distinct classes of proteins, one that binds FAD and another one that hydrolyzes FAD in the absence of the protein substrate of flavinylation (Fig. 2). This is consistent with the characterization of the two classes of purified Ftps shown in Figure 1. Active‐site residues are well conserved aside from the residue whose side chain contacts the isoalloxazine ring of the flavin. In all biochemically characterized Ftps that bind FAD, an aromatic residue (Tyr) is found near the flavin ring of FAD as opposed to a polar residue in the FAD pyrophosphatase Ftps (Fig. 2, see underlined residue number). The Tyr in the FAD‐bound Ftps forms a *π*‐stacking interaction with the isoalloxazine ring of bound FAD in the structure of *Salmonella enterica* Ftp (Ftp_Se) (Boyd et al. 2011). This suggests that the FAD hydrolytic activity of the Ftps is inhibited in the presence of a residue with an aromatic side chain that can pi‐stack with the isoalloxazine ring of FAD. To test this premise, the Tyr residue was altered to either an Ala or Asn residue.

**Figure 3:**
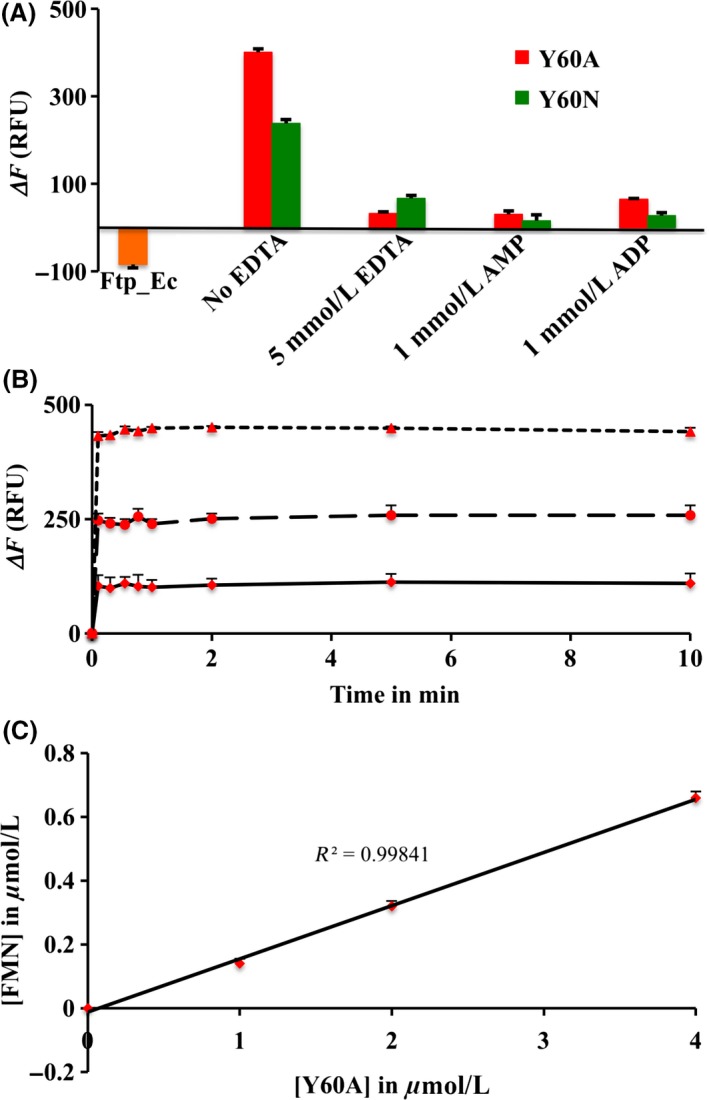
Functional characterization and reaction kinetics of wild‐type *E. coli* Ftp (Ftp_Ec) and its variants (*indicated in color*). (A) Mg^2+^‐dependent FAD pyrophosphatase activities of protein variants are shown. Under the reaction conditions, wild‐type Ftp_Ec (*in orange*) binds FAD as demonstrated by a decrease in the emission fluorescence. Data are presented as mean ± standard error (*n *=* *2). Standard error of the data points were <8 Δ*F*. (B) Single‐turnover kinetics showing the burst phase of the FAD pyrophosphatase reaction. Kinetics of the burst phase of the reactions was obtained by recording the fluorescence changes (Δ*F*) at room temperature, immediately after addition of the FAD substrate (10 *μ*mol L^−1^) to the preincubated enzyme reaction mixtures containing 1 (*solid line*), 2 (*long dashed line*) and 4 (*short dashed line*) *μ*mol L^−1^ of enzyme. Data are presented as mean ± standard error (*n *=* *2). Standard error of the data points were <25 Δ*F*. (C) Linear relationship between FMN formation and protein concentration during turnover. The points represent the FMN concentrations derived from the changes in fluorescence (Δ*F*) using an FMN standard curve (Deka et al. 2013a). Data are presented as mean ± standard error (*n *=* *2). Standard error of the data points were <0.02 *μ*mol L^−1^.

Earlier we reported that the recombinant Ftp protein of *E. coli* (Ftp_Ec) purified as a bright yellow protein and bound FAD with micro‐molar affinity (Deka et al. 2013a). However, no yellow color was associated either with the Y60A or Y60N variant of Ftp_Ec (Ftp_Ec_Y60A_ and Ftp_Ec_Y60N_). This led us to investigate the potential FAD hydrolytic activity of these variants. As shown in Figure 3A, both variants hydrolyzed FAD in the presence of Mg^2+^, and EDTA, AMP or ADP inhibited the hydrolytic activity. The observed FAD hydrolysis and inhibition are very similar to what has been reported for the Ftp_Tp Mg^2+^‐dependent FAD pyrophosphatase activity (Deka et al. 2013a).

To determine the catalytic competence of these protein variants, we also measured the extent and kinetics of FMN formation by the Ftp_Ec_Y60A_ protein variant; it exhibited burst kinetics with no steady‐state turnover (Fig. 3B and C) similar to what has been reported for Ftp_Tp (Deka et al. 2013a). As shown in Figure 3C, the protein variant generated ~0.2 *μ*mol of FMN per *μ*mol of enzyme at room temperature (~22°C). However, at higher temperature (37°C), 1 *μ*mol of protein variant enzyme generated ~400 relative fluorescence units (RFU) (Fig. 3A) which is equivalent to ~0.6 *μ*mol FMN (under reaction conditions 1 *μ*mol of FMN standard generates ~700 RFU; Deka et al. 2013a). This efficiency of FMN formation is similar to what has been reported for Ftp_Tp (Deka et al. 2013a), and is consistent with the single‐turnover kinetics of Mg^2+^‐dependent FAD pyrophosphatase catalyzed reactions. In this regard, the mechanistic property of the engineered enzyme was similar to the Mg^2+^‐dependent FAD pyrophosphatase of *T. pallidum*, since both are inhibited either by AMP/ADP or EDTA (Deka et al. 2013a) (Fig. 3A). Nonetheless, a single amino acid mutation (Ftp_Ec_Y60A_) leads to a switch in function from an FAD‐binding Ftp to a Mg^2+^‐dependent FAD pyrophosphatase Ftp. These results demonstrate that the Ftp_Ec_Y60A_ protein variant binds FAD, yet rapidly hydrolyzes it and the product FMN dissociates, resulting in the loss of yellow color. The loss of peaks in the UV–visible absorption spectrum due to FAD for this protein variant correlates with results reported for the analogous Y78A protein variant of *S. enterica* Ftp (Ftp_Se_Y78A_), although the authors interpreted these results as a total loss of FAD binding, as they did not assay the protein variant for FAD hydrolysis activity (Boyd et al. 2011). On the basis of these observations and the high sequence and structural similarity to the Ftp_Ec enzyme, we postulate that in fact the Ftp_Se_Y78A_ protein variant is also a Mg^2+^‐dependent FAD pyrophosphatase Ftp, similar to the Ftp_Ec_Y60A_ protein variant. The conversion of enzyme activity due to a point mutation is quite remarkable from a protein‐engineering point of view, and may have both functional and evolutionary implications for the two classes of Ftps.

### Evidence for Mg^2+^‐dependent redox protein flavinylation by Ftps

**Figure 4:**
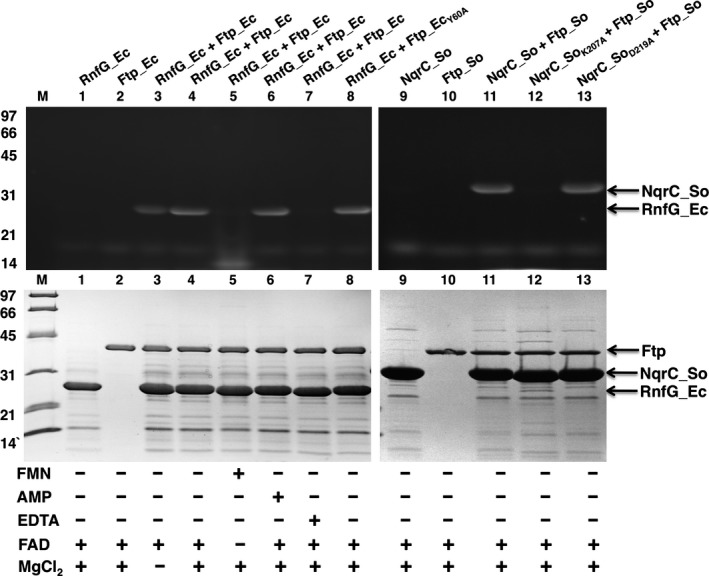
SDS‐PAGE characterization of flavinylation reactions. UV illumination of unstained gel is shown at the top, and Coomassie‐stained gel is shown below. Protein molecular mass markers are on the left side. Ftp_Ec (wild‐type and protein variants) and Ftp_So were reacted with their respective RnfG_Ec/NqrC_So (wild‐type and variants) under various conditions.

We have shown that there likely are two classes of Ftps, one associated with FAD‐binding and the other with FAD hydrolysis (Fig. 1). Recently it has been reported that the Ftp's of *Vibrio harveyi* (Ftp_Vh) and *Klebsiella pneumoniae* (Ftp_Kp) can flavinylate NqrC subunits of their respective Nqr‐redox systems via the covalent attachment of FMN to the threonine side chain found in an appropriate sequence motif (Bertsova et al. 2013). However, there is a paucity of information regarding Ftp‐mediated protein flavinylation in Rnf‐redox containing bacteria. As such, both NqrC and its RnfG homolog were employed in this study for further characterization of the metal‐dependent FMN transferase (flavinylation) reaction on a protein substrate. As mentioned earlier, recombinant RnfG_Ec and NqrC_So were purified as nonyellow apoproteins; they thus were used to study the in vitro flavin transferase activity of Ftp (Ftp_Ec and Ftp_So). As shown in Figure 4, recombinant wild‐type Ftp_Ec was able to flavinylate RnfG_Ec in a Mg^2+^‐dependent manner in the presence of FAD (lane 4), suggesting that this type of posttranslational flavinylation reaction indeed is a protein‐dependent FMN transferase activity, rather than an autocatalytic one. Although Ftp_Ec does not require additional Mg^2+^ to bind FAD in vitro (Deka et al. 2013a), its in vitro FMN transferase activity toward *E. coli* RnfG (RnfG_Ec) is enhanced upon the addition of exogenous Mg^2+^, and inhibited when treated with EDTA (Fig. 4, lane 7). Interestingly, the engineered protein variant (Ftp_Ec_Y60A_) with a Mg^2+^‐dependent FAD pyrophosphatase (EC 3.6.1.18) activity (Fig. 3A) also retained its Mg^2+^‐dependent FMN transferase (EC 2.7.1.180) activity on the protein substrate (Fig. 4, lane 8), indicating that the protein variant enzyme has dual activities (Deka et al. 2015). In addition, the FAD‐binding enzyme from an Nqr‐redox containing bacteria (Ftp_So) is able to flavinylate its cognate NqrC protein substrate in a Mg^2+^‐dependent manner (Fig. 4, lane 11). The combined results suggest that a single amino acid in Ftp likely differentiates its classes; however, both classes of Ftps (with either FAD‐binding or FAD pyrophosphatase activity (Fig. 2)) exhibit Mg^2+^‐dependent FMN transferase activity in the presence of a protein substrate.

### Structural characterization of the FAD‐binding FMN transferase Ftp_Ec reveals details about its bimetal active site

Our biochemical characterization has shown that Ftp_Ec can flavinylate its respective RnfG_Ec redox subunit in *E. coli* (Fig. 4). Although Ftp_Ec's FMN transferase activity on a protein substrate is metal‐dependent, nothing has been known regarding the nature and source of metal since no metal‐bound Ftp structure was employed in the above study. Therefore, we conducted extensive crystallization screening of Ftp_Ec wild‐type protein reconstituted in vitro with Mg^2+^ and FAD in an attempt to obtain a crystal structure of FAD and metal ions bound to the enzyme. However, we were only able to obtain crystals that were similar to the previously deposited structure (PDB ID: 2O18 (Seetharaman et al. 2006)), although at a higher resolution (1.88 vs. 2.20 Å) and a substantially improved R_free_ parameter at the end of model refinement (20.0 vs. 28.4%). We were also unsuccessful in our attempt to obtain an FAD‐bound structure of the Ftp_Ec_E169K_ protein variant (this residue is analogous to the active‐site residue K165 in Ftp_Tp), but once again obtained a structure that was determined at a higher resolution than wild‐type (1.75 vs. 1.88 Å) with a similarly improved final R_free_ parameter. There are four monomers in the asymmetric unit of each crystal structure, and the r.m.s.d. of the monomers within each structure ranges from 0.5 to 1.6 Å and 0.3 to 1.6 Å, respectively, between the two structures for approximately 300 C*α* carbons (Table 2).

**Table 2:** Ftp_Ec alignments.^1^

Structure	Monomer:Monomer		R.M.S.D.^2^ (Å)	#C‐alphas^3^
Ftp_Ec	A	B	1.6	301
	A	C	1.4	301
	A	D	0.5	304
	B	C	0.6	306
	B	D	1.3	299
	C	D	1.2	299
Ftp_Ec_E169K_	A	B	1.5	298
	A	C	1.5	299
	A	D	0.5	299
	B	C	0.6	304
	B	D	1.2	297
	C	D	1.1	297
Ftp_Ec_Y60N_	A	B	0.4	289
Ftp_Ec/Ftp_Ec_E169K_	A	A	0.3	304
	A	B	1.6	301
	A	C	1.4	301
	A	D	0.5	302
Ftp_Ec/Ftp_Ec_Y60N_	A	A	0.8	284
	A	B	0.9	287

^1^All alignments were performed using the Dalilite server ( http://www.ebi.ac.uk/Tools/structure/dalilite/).

^2^R.M.S.D., root mean squared deviation.

^3^Number of C‐alpha atoms aligned.

**Figure 5:**
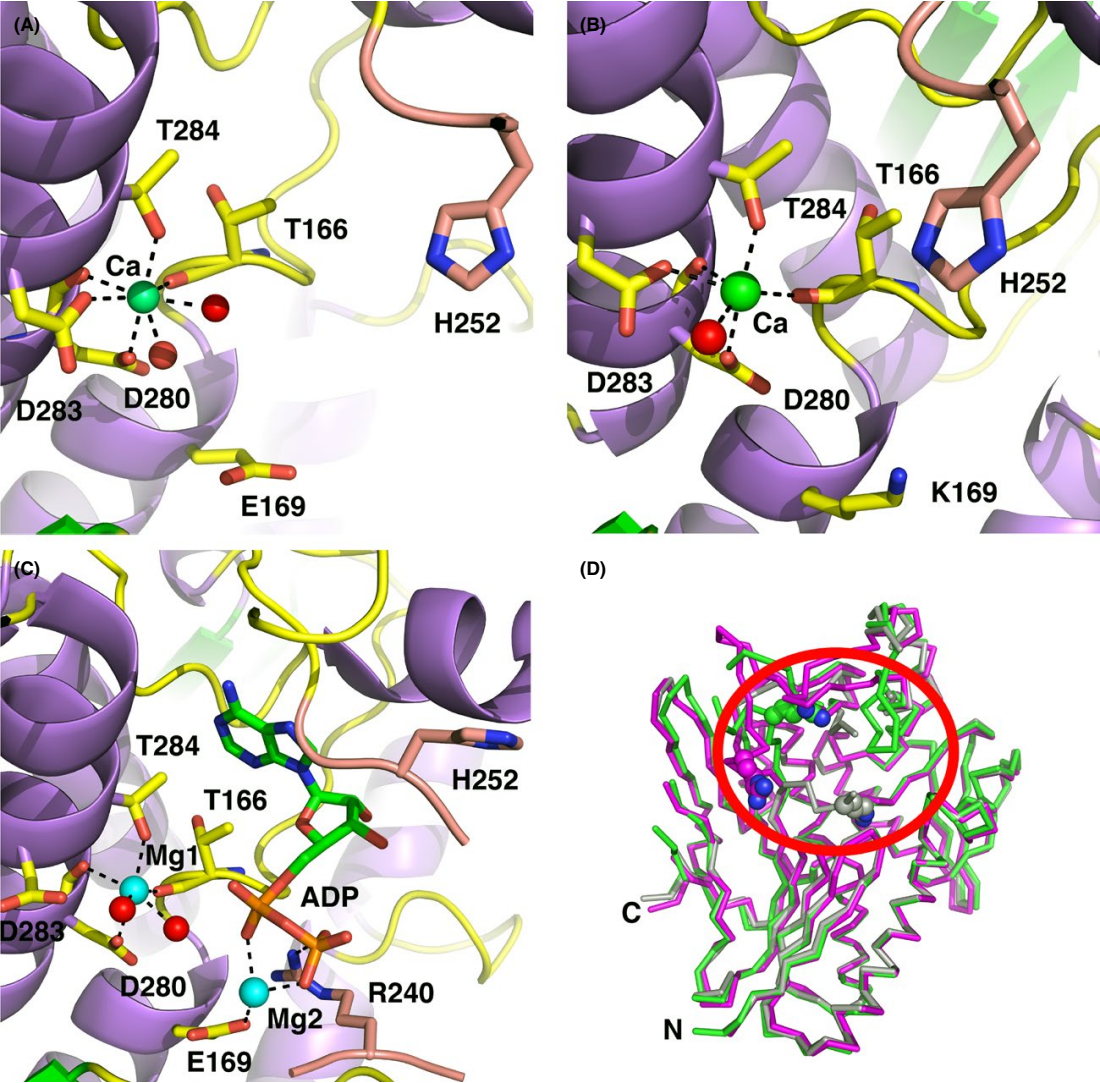
Active‐site geometries of Ftp_Ec structures. The carbon atoms of protein side chains are yellow, the carbon atoms from residues of the *β*‐hairpin insert are salmon, the nucleotides atoms are green, Ca^2+^ ions are green spheres, Mg^2+^ ions are cyan spheres, waters are red spheres, nitrogens are blue, and oxygens are red. Black dotted lines represent metal first‐coordination‐sphere contacts and important hydrogen bonding interactions. For clarity, some protein residues have been selectively removed from the images. (A) Apo wild‐type Ftp_Ec, monomer A. (B) Apo Ftp_Ec_E169K_, monomer A. (C) Ftp_Ec_Y60N_ Mg^2+^‐ADP complex, monomer A. (D) C*α* superposition of three representative Ftp_Ec monomer structures. Circled in red is the active site loop region (residues 247‐253) that displays variable conformations amongst the structures. Monomer A of Ftp_Ec wild‐type is colored green, monomer B of Ftp_Ec wild‐type is colored magenta, and monomer A of Ftp_Ec_Y60N_ is colored gray. Shown as a cyan sphere is the Ca^2+^ ion bound to metal site 1. Shown as color‐coded spheres is R240 in all three structures.

In the site previously denoted in the Ftp_Tp structure as metal binding site 1, a single metal ion is bound in the wild‐type and Ftp_Ec_E169K_ protein variant structures. Aided by the high resolution of our structural data, two water molecules were observed bound to each metal ion and thus the coordination number for the metal is seven, consistent with a Ca^2+^ rather than a Mg^2+^ ion as observed in the Ftp_Tp structures (Fig. 5A and B, Table 3). Refinement of the metal in this site as a Mg^2+^ ion resulted in large positive peaks in a difference electron‐density map; these peaks disappeared when modeled as a Ca^2+^ ion. Thus, in agreement with the original structure determination, we have assigned these as Ca^2+^ ions.

**Table 3:** Active site geometric parameters for *E. coli* Ftp structures

Crystal (monomer)	Apo, A	Apo, B	Apo, C	Apo, D	E169K, A	E169K, B	E169K, C	E169K, D	Y60N/ADP, A	Y60N/ADP, B
Resolution (Å)	1.88				1.75				1.90	
Me1 identity^1^	Ca^2+^	Ca^2+^	Ca^2+^	Ca^2+^	Ca^2+^	Ca^2+^	Ca^2+^	Ca^2+^	Mg^2+^	Mg^2+^
Me1 ave. B‐factor (Å^2^)	20.8	22.5	26.1	20.5	24.9	27.9	26.3	21.6	21.0	22.2
Me1 C.N.^2^	7	7	7	7	6	6	7	6	6	4
Me2 identity	**–**	**–**	**–**	**–**	**–**	**–**	**–**	**–**	Mg^2+^	Mg^2+^
Me2 ave. B‐factor (Å^2^)	**–**	**–**	**–**	**–**	**–**	**–**	**–**	**–**	39.0	38.8
Me2 C.N.	**–**	**–**	**–**	**–**	**–**	**–**	**–**	**–**	3	3
Bond distances (Å)										
Me1 – D280OD1	2.34	2.36	2.26	2.30	2.36	2.34	2.34	2.34	2.37	*3.35*
Me1 – D280O	2.31	2.35	2.28	2.28	2.29	2.28	2.26	2.25	2.49	2.56
Me1 – T166O	2.41	2.50	2.48	2.43	2.36	2.44	2.42	2.38	2.51	2.55
Me1 – T284OG1	2.42	2.38	2.44	2.46	2.39	2.35	2.40	2.39	2.42	2.50
Me1 – D283OD1	2.48	2.44	2.49	2.44	2.43	2.51	2.52	2.43	**–**	**–**
Me1 – H_2_O – 1	2.34	2.41	2.45	2.36	2.45	2.67	2.50	2.38	2.38	2.42
Me1 – H_2_O – 2	2.53	2.48	2.57	2.50	**–**	**–**	2.89	**–**	2.25	**–**
Ave. Me1 distances3	**2.40**	**2.41**	**2.43**	**2.40**	**2.38**	**2.43**	**2.48**	**2.36**	**2.40**	**2.51**
Me1 – Me2	**–**	**–**	**–**	**–**	**–**	**–**	**–**	**–**	*6.55* 4	*6.52*
Me2 – E169OE2	**–**	**–**	**–**	**–**	**–**	**–**	**–**	**–**	2.54	2.58
Me2 – *α*P_i_O	**–**	**–**	**–**	**–**	**–**	**–**	**–**	**–**	2.47	2.15
Me2 – *β*P_i_O	**–**	**–**	**–**	**–**	**–**	**–**	**–**	**–**	2.23	2.57
Ave. Me2 distances	**–**	**–**	**–**	**–**	**–**	**–**	**–**	**–**	**2.41**	**2.43**

^1^Me, metal.

^2^C.N., coordination number.

^3^Values in bold are averaged distances.

^4^Distances in italics are not assumed to represent first sphere metal ion coordination, but are included for reference.

As the Ftp_Ec_Y60N_ protein variant displays FAD pyrophosphatase activity that is inhibited by ADP, we obtained a crystal structure of the ADP complex with two metals bound (Fig. 5C). This structure provides the best model to date of the bimetal coordination of the ADP portion of an FAD substrate to this subclass of FMN transferases. In contrast to the apo structures, there are only two monomers in the asymmetric unit, and the r.m.s.d. of the monomers is 0.4 Å for 289 C*α* carbons (Table 3). The metal ion in site 1 in both monomers is no longer coordinated to a carboxylate oxygen atom from the side chain of D283, and thus has a coordination number of 6. Refinement of the metals as Ca^2+^ ions resulted in large negative electron density peaks that disappeared when modeled and refined as Mg^2+^ ions. Unlike the Ftp_Tp ADP‐inhibited structure, the *α*‐phosphate oxygens of the ADP do not coordinate to the metal ion in site 1. Coordinated to a second Mg^2+^ ion are *α*‐ and *β*‐phosphate oxygens of the ADP, as well as the side chain of E169. The side chain of R240 also coordinates to the *β*‐phosphate of the ADP. The distance between metal sites in this structure is over 6 Å, approximately twice the distance observed in the various Ftp_Tp inhibitor and substrate complexes, and we observed no metal‐coordinated water that can effectively bridge between the bimetal sites.

Despite the presence of a Ca^2+^ ion in metal site 1 in the apo enzyme, the inhibited Ftp_Ec_Y60N_ protein variant enzyme contains a bimetal Mg^2+^ center. It is reasonable to expect that the Ftp_Ec wild‐type enzyme, and by extension, the Ftp_Se enzyme, also utilize a bimetal site during the FMN transferase reaction. It is not clear if the Ftp_Ec wild‐type enzyme binds Mg^2+^ ions in both metal sites, or if a Ca^2+^ in metal site 1 and a Mg^2+^ in metal site 2 during the FMN transferase reaction. The metal coordination in the Ftp_Ec_Y60N_ protein variant/ADP structure suggests that the FAD pyrophosphatase reaction would require a Mg^2+^ ion in metal site 1 in order to activate the potential nucleophilic water attack on the ADP alpha‐phosphate. There are several examples of mixed Ca^2+^/Mg^2+^ bimetal sites in phosphoryl transfer enzymes, wherein the Ca^2+^ ion serves a role in properly positioning substrate and protein residues, whereas the Mg^2+^ ion is directly involved in catalysis (Beernink et al. 2001; Sudom et al. 2003; Kumaran et al. 2006). As the site of attack for the FMN transferase reaction is the *β*‐phosphate of the FAD, and given the large distance between the two metals in the ADP‐inhibited Ftp_Ec_Y60N_ structure, it is reasonable to expect that only metal site 2 would require a Mg^2+^ ion for this activity.

Superposition of three representative Ftp_Ec monomers (Fig. 5D) illustrates the inherent flexibility of the loop region near the active site comprising residues 237–253 (Table 2). Perhaps due to the relatively low solvent content of the crystals (approximately 42%), we do not observe this degree of flexibility in the Ftp_Tp structures, and access to the active site for substrate binding and product release is limited. The Ftp_Ec structures suggest that in solution the cores of the Ftp enzymes remain stable, whereas a single flexible loop adopts different conformations during the catalytic cycle.

### The structure of an Ftp‐mediated flavinylated protein, the NqrC subunit from *S. oneidensis*

**Figure 6:**
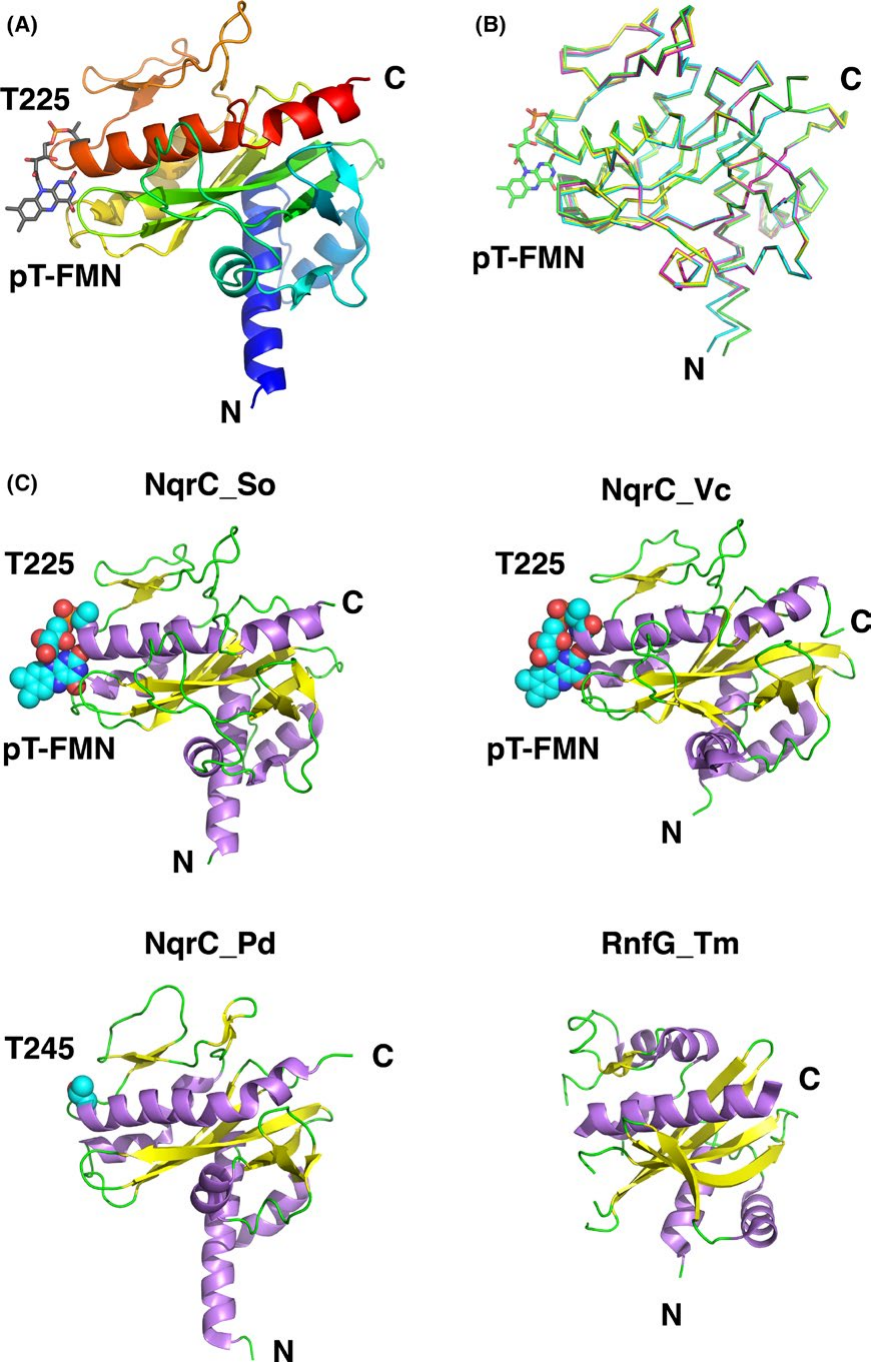
Analysis of the flavinylated NqrC_So structure. (A) The cartoon representation of the flavinylated NqrC_So protein (monomer A) is shown in colors of the rainbow starting with blue at the N‐terminus to red at the C‐terminus. Shown in stick representation and labeled is the phosphoester‐threonyl‐FMN residue (T225). (B) Superposition of all four monomers of the NqrC_So structure. Shown in stick representation and labeled is the phosphoester‐threonyl‐FMN residue (T225) from chain A. (C) NqrC structural homologs aligned and shown side‐by‐side for clarity. Clockwise from upper left, NqrC_So, this study; NqrC_Vc (PDB ID: 4U9S; Steuber et al. 2014); NqrC_Pd (PDB ID: 3LWX (Joint Center for Structural Genomics, 2010)); and RnfG_Tm (PDB ID: 3DCZ; Joint Center for Structural Genomics, 2011). Shown as cyan, blue, and red spheres are the atoms (carbon, nitrogen, and oxygen, respectively) of the phosphoester‐threonyl‐FMN residue (T225) in NqrC_So and NqrC_Vc, and the threonine target residue of flavinylation (T245) in NqrC_Pd. The threonine target residue of flavinylation (T202) in RnfG_Tm is disordered and was not modeled (Joint Center for Structural Genomics, 2011).

Prior to starting this study, there were no structurally characterized proteins with a phosphoester‐threonyl‐FMN (pT‐FMN) posttranslational modification (Heuts et al. 2009) and there was a dearth of information concerning how a pT‐FMN is formed on Ftp‐mediated flavinylated redox proteins (RnfG/NqrC). Despite extensive crystallization screenings, we were unable to obtain crystals of flavinylated RnfG_Ec (Fig. 4, lane 4). During the writing of this manuscript, the X‐ray crystal structure of the *Vibrio cholerae* Nqr complex and structures of several purified components of the complex were reported as well as the structure of a close NqrC homolog from *V. harveyi* (Steuber et al. 2014; Borshchevskiy et al. 2015). We obtained several crystal forms of the in vitro flavinylated *S. oneidensis* NqrC protein (Fig. 4, lane 11), expressed in a soluble version minus the N‐terminal membrane anchor, and phased the structure via the single‐wavelength anomalous dispersion method using the selenomethionyl‐substituted protein (Fig. 6A). There are four independent monomers of NqrC_So in the asymmetric unit, and the r.m.s.d. for superposition of these monomers ranges from 0.3 to 0.7 Å for over 214 C*α* atoms (Fig. 6B, Table 4). The fold of the protein resembles the NqrC structures from *Vibrio cholera* (NqrC_Vc, PDB ID: 4U9S and 4P6V; Steuber et al. 2014) and *Parabacteroides distasonis* (NqrC_Pd, PDB ID: 3LWX; Joint Center for Structural Genomics, 2010), and to the RnfG structure from *Thermatoga maritima* (RnfG_Tm, PDB ID: 3DCZ; Joint Center for Structural Genomics, 2011) (Fig. 6C). The r.m.s.d. for superposition of NqrC_So monomers to these structures ranges from 1.7 to 3.1 Å for 140 – 219 C*α* atoms (Table 4), with the best agreement to the NqrC_Vc isolated from the Nqr complex. Such large structural deviations to the homologous structures are probably responsible for our failure to phase the NqrC_So structure by molecular replacement, despite the almost 40% sequence identity of the Vibrio spp. proteins to NqrC_So. Part of the preceding loop and the target residue for flavinylation of the RnfG_Tm protein was disordered and not modeled in the structure determination; it is possible that this disordered region could become ordered in the flavinylated version of the protein.

**Table 4:** NqrC_So alignments.^1^

Structure	Monomer:Monomer		R.M.S.D.^2^ (Å)	#C‐alphas^3^
NqrC_So	A	B	0.7	220
	A	C	0.4	216
	A	D	0.5	215
	B	C	0.3	215
	B	D	0.3	214
	C	D	0.3	215
NqrC_So/NqrC_Vc (isolated)^4^	A	A	1.8	213
	B	A	1.7	212
	C	A	1.7	213
	D	A	1.7	213
NqrC_So/NqrC_Vc (in complex)^5^	A	A	2.3	219
	B	A	2.0	218
	C	A	1.9	214
	D	A	1.8	213
NqrC_So/NqrC_Pd^6^	A	A	2.7	192
	B	A	2.6	192
	C	A	2.6	187
	D	A	2.7	187
NqrC_So/RnfG_Tm^7^	A	A	2.9	140
	B	A	2.9	139
	C	A	3.1	140
	D	A	3.0	141

^1^All alignments were performed using the Dalilite server ( http://www.ebi.ac.uk/Tools/structure/dalilite/).

^2^R.M.S.D., root mean squared deviation.

^3^Number of C‐alpha atoms aligned.

^4^PDB ID: 4U9S.

^5^PDB ID: 4P6V.

^6^PDB ID: 3LWX.

^7^PDB ID: 3DCZ.

**Figure 7:**
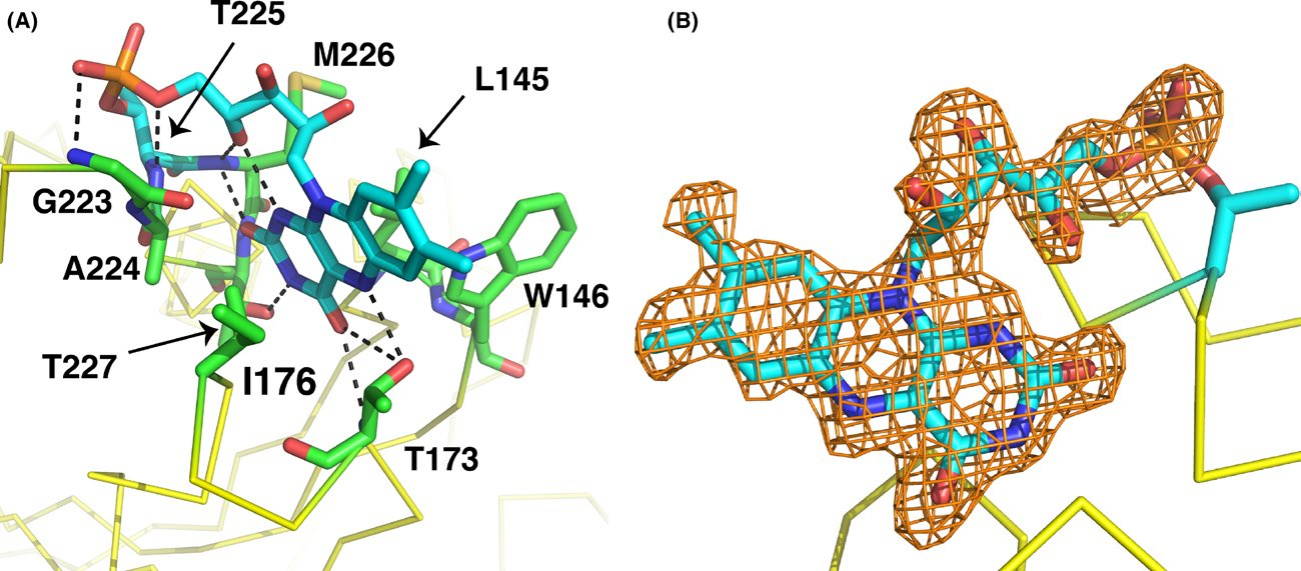
Flavin site geometry of NqrC_So structure. (A) Shown in stick representation with green carbon atoms are residues involved in interactions with the phosphoester‐threonyl‐FMN residue, which is shown with cyan carbons. (B) Omit electron density around the FMN portion of the pT‐FMN ligand. Shown in orange mesh is the |mFo – DFc| omit electron density, contoured at the 2.5*σ* level and superimposed on the refined structure coordinates. This map was calculated by omitting the FMN portion of the pT‐FMN ligand from the model and conducting three rounds of maximum‐likelihood positional and B‐factor refinement.

**Figure 8:**
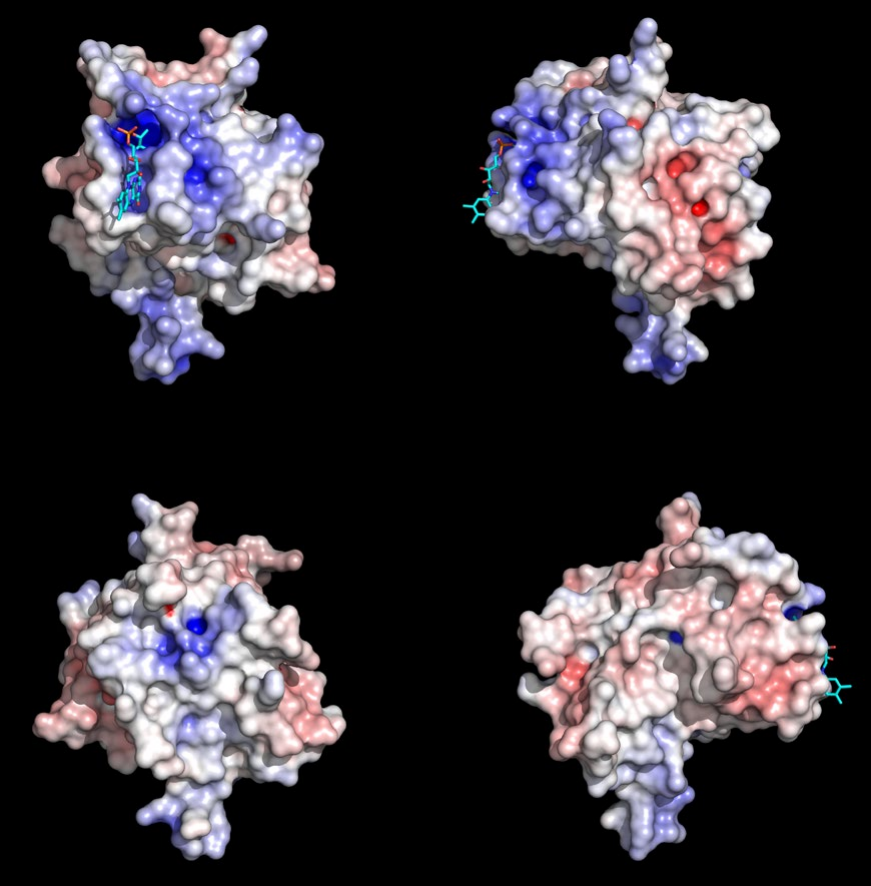
Electrostatic potential of the flavinylated NqrC_So structure. Surface representations illustrating the electrostatic potential of the NqrC_So structure; shown is monomer A. The view in the upper right quadrant of the panel is the same as shown in Fig. 6A, and proceeding clockwise, the other three views are 90° rotations around the vertical axis. All electrostatic potentials were calculated with the APBS (Baker et al. 2001) module implemented in PyMOL (Schrodinger 2010), and the gradients shown ranged from ≥−10 kBT/e (red) to ≤10 kBT/e (blue).

**Figure 9:**
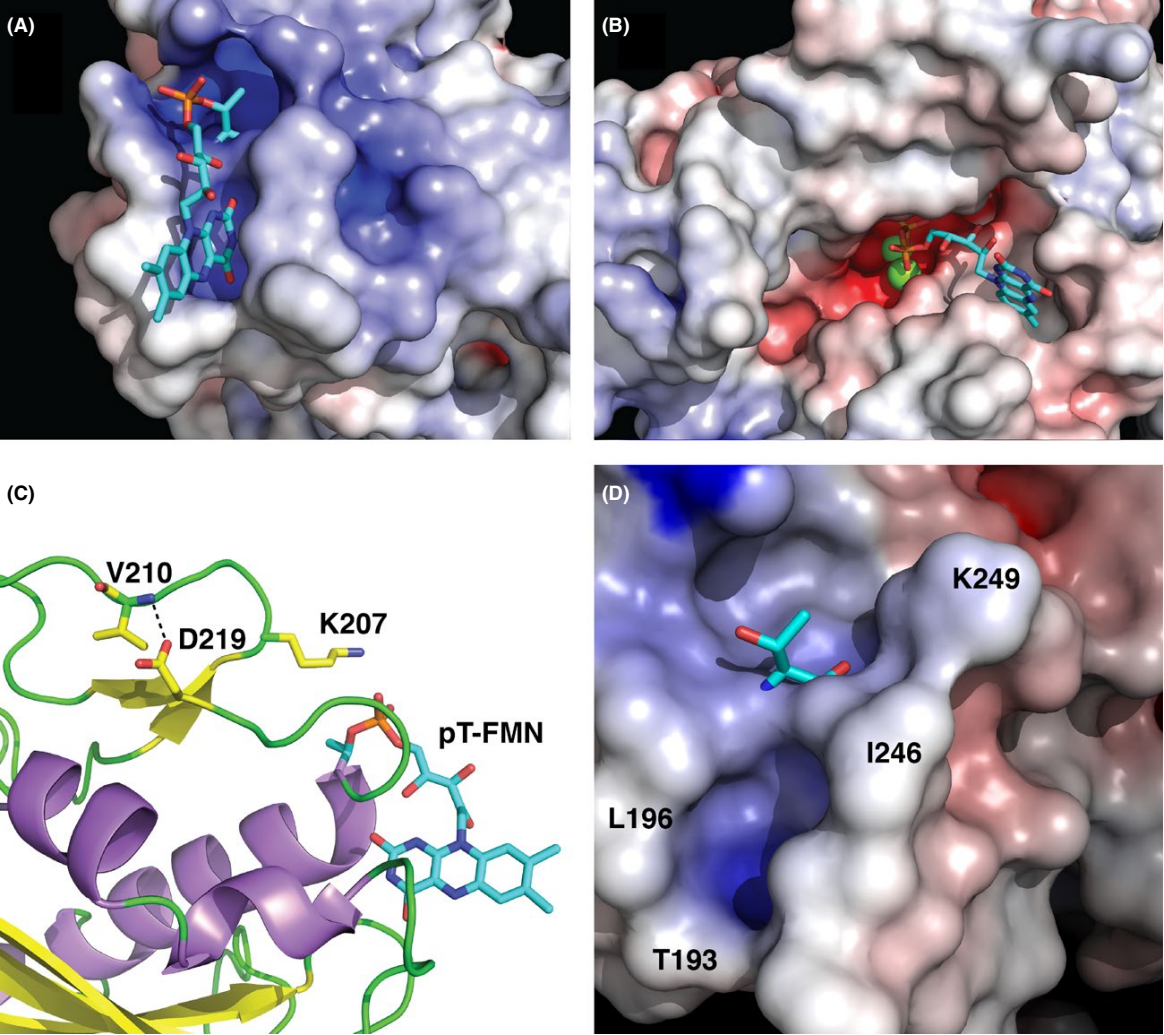
FMN binding site analysis. Zoomed‐in view of surface representations illustrating the electrostatic potential of (A) the NqrC_So flavinylation target site and (B) the Ftp_Tp active site (Deka et al. 2013a). Shown in stick representations are the phosphoester‐threonyl‐FMN residue in NqrC_So and the bound FAD in Ftp_Tp, and Mg^2+^ ions are shown as green spheres. (C) Shown with yellow carbon atoms in stick representation are residues that participate in the hydrogen bond between the side chain of the highly conserved D219 and the main chain amide in the loop that caps the phosphoester‐threonyl‐FMN binding site. Also shown in this format is the side chain of the conserved K207 residue. (D) Zoomed‐in view of a surface representation illustrating the electrostatic potential of the NqrC_Pd flavinylation target site. Shown in stick representation is T245, and selected residues that line the FMN binding cleft are labeled.

The modified threonine (T225) lies at the N‐terminal end of the C‐terminal alpha helix of NqrC_So, in a groove formed by a number of conserved protein side chains (Fig. S1). The pyrimidine moiety of the isoalloxazine ring of the FMN makes extensive hydrogen‐binding and hydrophobic contacts with protein residues in the groove (Figs. 7A and B, Fig. S2), and the dimethylbenzene moiety points outwards toward the surface of the protein, in good agreement with the NqrC_Vc and NqrC_Vh structures (Steuber et al. 2014; Borshchevskiy et al. 2015). A PROMALS3D structure‐based sequence alignment (Pei et al. 2008) of several representative bacterial NqrC and RnfG proteins (Fig. S1) reveals higher sequence conservation for residues that make hydrophilic contacts with the FMN than for residues that make hydrophobic contacts. Surface representations illustrating the electrostatic potential of the NqrC_So structure (Fig. 8) reveal the high solvent exposure of the phosphoester‐threonyl‐FMN residue (47% solvent accessible surface exposure for the threonine side chain; 55% exposure for the isoalloxazine ring of the FMN; 49% exposure for the remaining atoms of the FMN). An electrostatic potential calculation of the NqrC_So flavinylation target site (Fig. 9A) shows that the environment in the binding cleft for the FMN is positively charged. In contrast, the Ftp_Tp active site (Fig. 9B) is negatively charged, and the bound FAD is more buried from the solvent (solvent accessible surface exposure of the AMP portion of the FAD is ~1.5%, and the isoalloxazine ring is ~31%; Deka et al. 2013a). The orientation of the isoalloxazine ring bound in the Ftp_Tp structure, which has the hydrophilic end of the ring pointing toward the solvent, is opposite to that seen in the flavinylated NqrC_So. This orientation is consistent with the presumed nucleophilic attack of an NqrC threonine residue on the diphosphate moiety of the Ftp‐bound FAD as a first step toward FMN transfer. Given the remarkably high flexibility observed for residues 237–253 in the Ftp_Ec structures, it is likely that in solution the Ftp enzyme active site undergoes a structural rearrangement to allow this attack of the flavinylation target protein threonine to occur.

### Identification of an essential lysine residue and functional implication of the phosphoester‐threonyl‐FMN center of NqrC_So

One of the highly conserved residues in the NqrC/RnfG proteins is K207 (Fig. S1). The side chain of this residue is located near the nucleophilic T225 and the phosphate of the pT‐FMN modification (Fig. 9C). In analogy to the mechanism of Class A *β*‐lactamases and penicillin‐binding proteins, we postulate that this lysine is ideally situated to support the deprotonation of the threonine hydroxyl, thereby activating it as a suitable nucleophile for the transferase reaction (Golemi‐Kotra et al. 2004; Zhang et al. 2007). In solution at physiological pH of 7.5, this lysine in the isolated NqrC protein is solvent‐exposed and protonated (O'Neil 2006), but in complex with its cognate Ftp protein, the lysine would be buried from the solvent in the FAD binding pocket. Interaction with the negatively charged phosphate of FAD may abstract a proton to yield the free‐base form of the conserved lysine, which would reduce the pKa sufficiently to allow deprotonation of the T225 hydroxyl. Another one of the highly conserved residues in the NqrC/RnfG proteins is D219. Inspection of the NqrC_So structure reveals that this residue is not part of the FMN‐binding cleft, but is in a nearby beta strand and forms a hydrogen bond to the backbone amide of the loop that contains K207 (Fig. 9C). This hydrogen bond appears to stabilize the loop and thus properly position K207 near the site of flavinylation, T225. This interaction is conserved in the Nqrc_Vc and NqrC_Pd structures, but not the RnfG_Tm structure, perhaps due to the 17 disordered residues of the putative FMN binding cleft. Analysis of a surface representation illustrating the electrostatic potential of the NqrC_Pd structure reveals a cleft near the flavinylation target, T245 (Fig. 9D), that is similar in electrostatic properties and size to the FMN binding cleft of the NqrC_So protein. Thus, the FMN binding cleft appears to be well ordered in some NqrC/RnfG proteins even in the absence of the posttranslational modification.

To test the hypothesis of essentiality of the residues identified above to the flavinylation reaction, we mutated them individually to alanine. The K207A mutation exhibited no detectable in vitro flavinylation activity (Fig. 4, lane 12), confirming the hypothesis of essentiality. However, the D219A variant had in vitro activity similar to that of the wild‐type protein (Fig. 4, lane 13). While it is possible that the side chain D219 is inessential for the positioning of K207, it is also feasible that the absence of D219′s carboxylate moiety is compensated for by alternative interactions. Further investigation is required to clarify the role of D219 in the flavinylation reaction. The identification of K207 as a residue critical for flavinylation is highly significant; despite previously published extensive mutagenesis of Ftp_Tp, the only residues identified as critical for FMN transferase activity were conserved residues that ligate the bound Mg^2+^ ions in the enzyme (Deka et al. 2015).

The high solvent accessibility of the phosphoester‐threonyl‐FMN residue appears unusual, given that this cofactor is part of an electron‐transfer chain in a multi‐subunit integral membrane complex. In the NqrC_So crystalline lattice, the cofactor is shielded from the bulk solvent by symmetry‐related molecules. In the *V. cholerae* Nqr complex, the hydrophobic end of the isoalloxazine ring of the FMN is buried in a cleft formed by the NqrD and NqrE subunits and thus shielded from the bulk solvent (Steuber et al. 2014).

**Figure 10:**
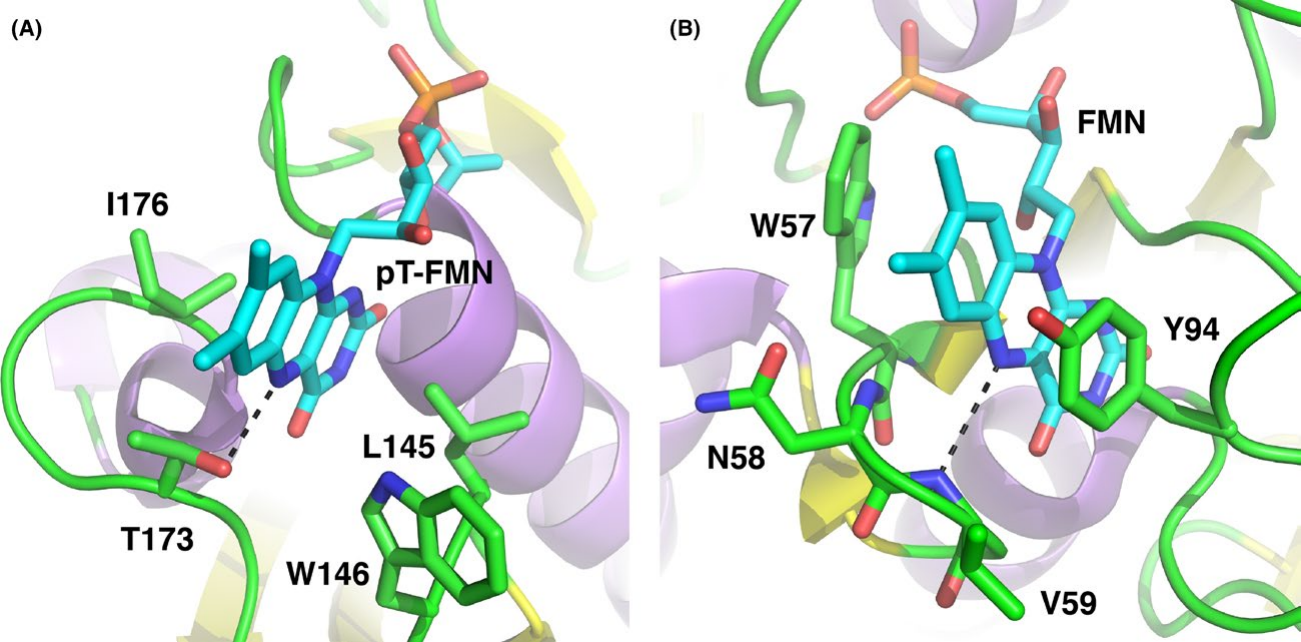
Comparison of FMN binding sites in NqrC_So and flavodoxin. (A) The pT‐FMN modification in NqrC_So (current work). (B) The fully oxidized form of *Anacystis nidulans* flavodoxin (PDB ID: 1CZN; Drennan et al. 1999). For each protein, the dashed line shows the hydrogen‐bonding interaction between the N5 of the isoalloxazine ring and a loop of the protein.

The coordination of the pT‐FMN residue (Fig. 10A) bears a striking resemblance to that observed in the flavodoxin family of electron transfer proteins (Fig. 10B) (Sancho 2006). Flavodoxins bind FMN noncovalently and utilize this cofactor to cycle between oxidized and fully reduced (hydroquinone) forms via an intermediate partially reduced (flavosemiquinone) state. The isoalloxazine ring of flavodoxins is typically buried in a hydrophobic pocket, sandwiched between aromatic residues such as tryptophan and tyrosine, with the dimethylbenzene portion of the conjugated ring exposed to the solvent. The highly hydrophobic nature of the binding pocket favors the reduced state of the FMN, thus modulating the reduction potential to more negative values than observed for free FMN in solution (Sancho 2006). In the oxidized state, the N5 atom of the isoalloxazine ring hydrogen bonds to a main chain amide in a *β*‐turn, whereas in the singly reduced flavosemiquinone state, the isoalloxazine ring remains relatively planar and the N5 atom is typically protonated. This causes a rearrangement of the *β*‐turn to provide a hydrogen bond acceptor (e.g., a backbone carbonyl oxygen) to interact with the N5(H) (Hoover et al. 1999; Kasim and Swenson 2000). The binding pockets for the pT‐FMN cofactor in NqrC_So and in NqrC_Vc are less hydrophobic than typically found in flavodoxins, yet these proteins also function as electron‐transfer proteins and share a similar hydrogen‐bonding interaction of the pT‐FMN N5 atom to a threonyl side chain O*γ* atom in a protein loop. EPR and ENDOR spectroscopy on *Vibrio cholerae* Na^+^‐Nqr pumps that contain wild‐type and protein variant NqrB and NqrC subunits suggest that the FMN cofactors in these proteins are stable in the one‐electron reduced (flavosemiquinone) forms, and are anionic (not protonated) (Barquera et al. 2006). Further investigations will reveal whether the reduction in the pT‐FMN cofactor in NqrC_So or in the *V. cholerae* Nqr complex results in protein backbone rearrangements similar to that seen in flavodoxins.

### Biological significance of Ftp and posttranslational protein flavinylation

Flavoproteins are known to take part in a large number of cellular processes. In addition to their role as redox catalysts, flavins are also found in some nonredox enzymes, such as transferases, isomerases, ligases, and lyases (Macheroux et al. 2011; Deka et al. 2013a). Although almost 90% of flavin‐enzymes contain noncovalently bound flavins (Macheroux et al. 2011), there is a small group of enzymes where the flavin ring is covalently linked to an amino acid residue such as Cys, Tyr, Thr, or His (Heuts et al. 2009). Most covalent flavin attachment is thought to be posttranslational and autocatalytic; in this regard, the current findings not only highlight the role of metal‐dependent Ftp catalysis in the phosphoester‐threonyl‐FMN bond formation in the posttranslationally modified redox proteins in both Rnf and Nqr redox containing bacteria, but also underscore the periplasm as a metabolically active subcellular compartment for flavoprotein biogenesis. Also, in bacteria, flavin‐based ion motive forces (Na^+^/H^+^‐Rnf/Nqr) are coupled to many physiological processes including ATP synthesis, rotation of the flagellar motor, and accumulation of nutrients that are taken up by symporters (Biegel et al. 2011; Mayer and Muller 2014; Deka et al. 2015). Because periplasmic posttranslational flavinylation is a prerequisite for the redox‐carrying functions of the Rnf and Nqr subunits (RnfG and NqrC), Ftp's metal‐dependent activity is critical to the biologically active Rnf/Nqr redox pump, which is central to both cytoplasmic membrane redox and overall bioenergetics in many pathogenic bacteria. Although Ftp's metal‐dependent protein flavinylation (Mg^2+^‐FMN transferase) was the major focus of our study, Ftp's role in the periplasm likely is not restricted to flavoprotein biogenesis, because some Ftp orthologs bind FAD (Fig. 1A) and others hydrolyze it into AMP and FMN (Fig. 1B) in the absence of a protein substrate. The latter activity probably is necessary to maintain the periplasmic flavin pool (Deka et al. 2013a, 2015). Finally, in *Shewanella oneidensis* it is believed that FAD synthesized in the cytoplasm is exported across the cytoplasmic membrane by an unidentified mechanism, where FAD is then processed for extracellular flavin electron shuttles (Covington et al. 2010). This study raises the possibility that exported FAD in the *Shewanella* periplasm could be utilized by an FAD‐binding type of Ftp (e.g., Ftp_So; Fig. 1A) for its transfer onto a redox protein such as NqrC_So, via its metal‐dependent FMN transferase (Fig. 4 and Fig. 6). This scenario is predicated on the demonstrated potential functional diversity within the Ftp family.

This study expands our comprehension of the role of the bacterial periplasm as a metabolically active compartment, not only for flavoprotein biogenesis, but also for overall membrane redox homeostasis. The sustaining function of a cytoplasmic membrane redox system (Rnf/Nqr) depends on periplasmic flavin and the activity of flavin‐trafficking protein (Ftp). Although we do not know the periplasmic source of flavin or why bacteria have evolved posttranslational flavinylation in the periplasm, our structural and biochemical analyses provided some new insights regarding how metal‐dependent flavinylation occurs between a flavin donor (Ftp) and a flavin‐acceptor protein (e.g., RnfG/NqrC). However, additional biophysical, biochemical, and biological studies of the interactions between Ftp (flavin donor) and RnfG/NqrC proteins (flavin acceptors) are warranted to better comprehend the mechanism(s) of posttranslational protein flavinylation. Finally, it is not implausible that the catalytic core of Ftp may prove useful as a new platform for structure‐based drug discovery of nontraditional broad‐spectrum antimicrobials that kill bacterial pathogens without harming the human host.

## Conflict of Interest

None declared.

## Supporting information


**Figure S1.** PROMALS3D (1) structure‐based sequence alignment of NqrC and RnfG flavinylation target proteins.
**Figure S2.** Flavin site geometry of NqrC_So structure.
