# Supplementary material for: Molecular insights into the enzymatic diversity of flavin‐trafficking protein (Ftp; formerly ApbE) in flavoprotein biogenesis in the bacterial periplasm
Source: Microbiologyopen. 2015 Dec 2;5(1):21–38. doi: 10.1002/mbo3.306 (PMC4767422; doi:10.1002/mbo3.306)
Supplement: Supplementary file 1 — Figure S1. PROMALS3D (1) structure‐based sequence alignment of NqrC and RnfG flavinylation target proteins. Figure S2. Flavin site geometry of NqrC_So structure. [file MBO3-5-021-s001.pdf]

**Figure S1. PROMALS3D (1) structure-based sequence alignment of NqrC and RnfG flavinylation target proteins.** The first line in each block shows conservation indices for positions with a conservation index above 5. Each representative sequence has a magenta name and is colored according to PSIPRED (2) secondary structure predictions (red: alpha-helix, blue: beta-strand). A representative sequence and the immediate sequences below it with black names, if there are any, form a closely related group. The consensus amino acid sequence (Consensus_aa) and consensus predicted secondary structures (Consensus_ss) are labeled. Consensus predicted secondary structure symbols: alpha-helix: h; beta-strand: e. Functionally important conserved residues are highlighted in yellow and labeled above the sequence alignment with the following codes: residues that contact the FMN in the NqrC_So structure are labeled with an asterisk (*); the putative catalytically important lysine residue is labeled with a dagger (†); and the conserved hydrogen-bonding residue in the loop preceding the threonine target of flavinylation is labeled with an ampersand (&).

**Figure S2. Flavin site geometry of NqrC_So structure.** Ligplot+ (3) schematic of residues involved in interactions with the phosphoester-threonyl-FMN residue. The bonds of the FMN modification are purple, the bonds of protein side chains are brown, carbon atoms are black spheres, nitrogens are blue, oxygens are red, and sulfurs are yellow. Green dotted lines represent hydrogen-bonding interactions with interatomic distances labeled. Hydrophobic interactions of protein residues with the FMN ligand are represented as red arcs with the name of the residue labeled. Corresponding atoms of both ligand and protein residues involved in hydrophobic contacts are shown with a halo of short red lines radiating from the atom sphere.

1. **Pei J, Kim BH, Grishin NV.** 2008. PROMALS3D: a tool for multiple protein sequence and structure alignments. Nucleic Acids Res **36:**2295-2300.
2. **Jones DT.** 1999. Protein secondary structure prediction based on position-specific scoring matrices. J Mol Biol **292:**195-202.
3. **Laskowski RA, Swindells MB.** 2011. LigPlot+: multiple ligand-protein interaction diagrams for drug discovery. J Chem Inf Model **51:**2778-2786.

**Figure S1.**

```
Conservation:                 8
RnfG_Tm                    1  M----------------------------------------------KDILKTGLILMVFTAISGLFLGL   24
RnfG_Tm coords.               ----------------------------------------------------------------------
NqrC_Pd                    1  MAKYKCKVCGYVHEGNKAPDTCPVCSAPASEFEEIKEEGAAKKGINRDSNVYTVVYAAVMVVLVAVVLAF   70
NqrC_Pd coords.               ----------------------------------------------------------------------
NqrC_Vc                    1  MAS------------------------------------------NNDSIKKTLFVVIALSLVCSIIVSA   28
NqrC_Vc coords.               ----------------------------------------------------------------------
NqrC_Vh                    1  MAS------------------------------------------NNDSIKKTLGVVIGLSLVCSIIVST   28
NqrC_Hd                    1  MAK-----------------------------------------FNKDSVSGTLTVVVLLSLICSLIVAS   29
NqrC_So                    1  MVF------------------------------------------KKDTVVGTMIFTITLCLLCSFMITG   28
NqrC_So coords.               ----------------------------------------------------------------------
NqrC_Td                    1  M----------------------------------------------KQMIKLALTLSAYAVIACLALAA   24
RnfG_Vc                    1  ML---------------------------------------------TAIRKNGLILAVFACVSTGLVAL   25
RnfG_Ec                    1  ML---------------------------------------------KTIRKHGITLALFAAGSTGLTAA   25
NqrC_Ef                    1  MK--------------------------------------------------VNK----------FVKGF   10
Consensus_aa:                 M.....................................................................
Consensus_ss:                                                                   hhhhhhhhhhhhhhhhhhhh
```

```
Conservation:                     5   5              5
RnfG_Tm                   25  VYVGVKGKIQEADNAAKLSAIKFVLKDPLTGDYLVDEKE----IEEIVKKT-----------------GI   73
RnfG_Tm coords.           37  ------------DNAAKLSAIKFVLKDPLTGDYLVDEKE----IEEIVKKT-----------------GI   73
NqrC_Pd                   71  TSQSLRSFQKQNEDNDKRQQILRSINVNVSS---SEAETKYNELIKEAFLVNENGEKVE--GD---AFAT  132
NqrC_Pd coords.           73  --QSLRSFQKQNEDNDKRQQILRSINVNVSS---SEAETKYNELIKEAFLVNENGEKVE--GD---AFAT  132
NqrC_Vc                   29  AAVGLRDKQKENAALDKQSKILQVAGIEAKGS--KQIVELFNKSIEPRLVDFNTGDFVE--GD---AANY   91
NqrC_Vc coords.           33  ----LR---------DKQSKILQVAGIEAKGS--KQIVELFNKSIEPRLVDFNTGDFVE--GD---AANY   91
NqrC_Vh                   29  AAVGLRDKQKANAVLDKQSKIVEVAGIEADG---KKVPELFAEYIEPRLVDFKTGDFVETAGDGSTAANY   95
NqrC_Hd                   30  AAVLLKPTQDIQKQLDKQKNILQAAGLMHEN---TNVQETYAKFIEPKIVDLATGDYVE--D----VANF   90
NqrC_So                   29  TAGVLKERKLAKKRDELQRYVLMAADVNLGQG--NEFRDIFAKSVKPLLINLDTGKVDS--DAN--VLDF   92
NqrC_So coords.           32  ---SLKERKLAKKRDELQRYVLMAADVNLGQG--NEFRDIFAKSVKPLLINLDTGKVDS--DAN--VLDF   92
NqrC_Td                   25  VYNFTAPRIAEVKIEKTNRALKAVFPEAEDF---KEIS--------------------------------   59
RnfG_Vc                   26  TYALTAEQIQQQEQKQLLQVLNQVIPHKYHD---NPLA--------QACTLV------------------   66
RnfG_Ec                   26  INQMTKTTIAEQASLQQKALFDQVLPAERYN---NALA--------QSCYLV------------------   66
NqrC_Ef                   11  AAIALSSL------------VLAACGADKKD-----------------------NTTNS--SS---AASS   40
Consensus_aa:                 ....hp..b......pb.p.l..hhs...p....pph...............................sh
Consensus_ss:                 hhhhhhhhhhhhhhhhhhhhhhhh           hhhh       hh
```

```
                                                                                     **
Conservation:                 6                               55 5                  6  5          5
RnfG_Tm                  74   ETVV-------LKE----YKEG-----VVLGPLYEFVTKD-GRNAYVLSG-YAPGFGGNVTVVACFIKTE   125
RnfG_Tm coords.          74   ETVV-------LKE----YKEG-----VVLGPLYEFVTKD-GRNAYVLSG-YAPGF-GNVTVVACFIKTE   125
NqrC_Pd                 133   DVVKAA--------------------TEHQYPVFVANVDG--QPKYIMAL-HGAGLWGPLWGYISVDSD-   178
NqrC_Pd coords.         133   DVVKAA--------------------TEHQYPVFVANVDG--QPKYIMAL-HGAGLWGPLWGYISVDSD-   178
NqrC_Vc                  92   DQRKAAKEASESIKLTAEQDKAKIQRRANVGVVYLVKDGD-KTSKVILPV-HGNGLWSMMYAFVAVETD-   158
NqrC_Vc coords.          92   DQRKAAKEASESIKLTAEQDKAKIQRRANVGVVYLVKDGD-KTSKVILPV-HGNGLWSMMYAFVAVETD-   158
NqrC_Vh                  96   DQRKAAKDPAESIKLTAEEDKAKILRRANTGIVYLVKSGD-EVSKVIIPV-HGTGLWSMMYAFVAVETD-   162
NqrC_Hd                  91   DAKAFAKDPATSVAIKPEDDKANIRMRAKYAEVYLVKDEMGQTTQVVLPM-YGNGLWSMMYGFVAVQPD-   158
NqrC_So                  93   DERMAAINPETSSTPK--KDIAKIKTRANDARVFKVFDDSGKLSSVVVPF-YGKGLWSMIYGYVAVEPD-   158
NqrC_So coords.          93   DERMAAINPETSSTPK--KDIAKIKTRANDARVFKVFDDSGKLSSVVVPF-YGKGLWSMIYGYVAVEPD-   158
NqrC_Td                  60   ------------------AEIPEGLNKTTFLNAYTAIKNG-KTVGLTITA-KGPTYAS-ATILIAMDLN-   107
RnfG_Vc                  67   --------------------NDDKLGTAKTMHAYLAQRDG-QPTAIAIETIAPDGYNGEIKLIVGIANN-   114
RnfG_Ec                  67   --------------------TAP-ELGKGEHRVYIAKQDD-KPVAAVLEATAPDGYSGAIQLLVGADFN-   113
NqrC_Ef                  41   ETKK---------------------STESSAPAKKVAGGDLKDGTYKLEE-KNEKNGYRAVFEMTVKD--    86
Consensus_aa:                 -....................s....p.....h@.h...s.p.s.hhl....s.sh.t.hh.hhth..s.
Consensus_ss:                  hh                            eeeeeee    eeeeeeee  e     eeeeeeee


                                              *  *
Conservation:                    65    5     676667 5                      5 6
RnfG_Tm                 126   DGFMLNSVRVIDYSQETPGLGAKIG-------------EESIQRRFFPVPPEGLKN--------------   168
RnfG_Tm coords.         126   DGFMLNSVRVIDYS------------------------QESIQRRFFPVPPEGLKN--------------   168
NqrC_Pd                 179   -KNTIYGADFSHQG-ETPGLGAEIS-------------KPVFSNEFKGKKIFMSGEF-------------   220
NqrC_Pd coords.         179   -KNTIYGADFSHQG-ETPGLGAEIS-------------KPVFSNEFKGKKIFMSGEF-------------   220
NqrC_Vc                 159   -GNTVSGLTYYEQG-ETPGLGGEVE-------------NPAWRAQWVGKKLFDENHK-------------   200
NqrC_Vc coords.         159   -GNTVSGLTYYEQG-ETPGLGGEVE-------------NPAWRAQWVGKKLFDENHK-------------   200
NqrC_Vh                 163   -GDTVSGITYYEQG-ETPGLGGEVE-------------NPAWRAQFVGKKLFDENHK-------------   204
NqrC_Hd                 159   -ANTVNGITYYEQG-ETAGLGGEIA-------------NPNWQKSFVGKKLFNANNE-------------   200
NqrC_So                 159   -FNTIKGVVVYEHG-ETPGIGDFVT-------------DPHWLSLWKGKQLFDDKGK-------------   200
NqrC_So coords.         159   -FNTIKGVVVYEHG-ETPGIGDFVT-------------DPHWLSLWKGKQLFDDKGK-------------   200
NqrC_Td                 108   --KTIRKIEFLELT-DTPSLGSKAA-------------DEPFAGQFNGKALDSAF---------------   146
RnfG_Vc                 115   -G-TVLGVRVLAHQ-ETPGLGDKIDLR-----------ISNWVLGFNGQQVTADNQ--------------   156
RnfG_Ec                 114   -G-TVLGTRVTEHH-ETPGLGDKIELR-----------LSDWITHFAGKKISGADD--------------   155
NqrC_Ef                  87   -G-KITESKYDNIN-ADGKSKTEDTKYEESMKAKSGVGPKEYIKQLNDSFVKAQSASGVEVVTGATHSSE   153
Consensus_aa:                 ...hl..hph.p.s.-TstlGsch.................@..p@.sp.l.s.................
Consensus_ss:                    eeeeeeeeee                          hhhhhh
```

```
                                                                               †
Conservation:                                                                  7
RnfG_Tm              169  ------------------------------------------------GLRVDKDAG-------------  177
RnfG_Tm coords.      169  ------------------------------------------------GLRVDKDAG-------------  177
NqrC_Pd              221  -----------------------------------------------KSVAVVK----------------  227
NqrC_Pd coords.      221  -----------------------------------------------KSVAVVK----------------  227
NqrC_Vc              201  -----------------------------------------------PAIKIVK--G-------------  208
NqrC_Vc coords.      201  -----------------------------------------------PAIKIVK--G-------------  208
NqrC_Vh              205  -----------------------------------------------PAIKIVK--G-------------  212
NqrC_Hd              201  -----------------------------------------------VALTIGK--G-------------  208
NqrC_So              201  -----------------------------------------------FAMRLVK--G-------------  208
NqrC_So coords.      201  -----------------------------------------------FAMRLVK--G-------------  208
NqrC_Td                   ----------------------------------------------------------------------
RnfG_Vc              157  -----------------------------------------------DDWKVRK----------------  163
RnfG_Ec              156  -----------------------------------------------AHWAVKK----------------  162
NqrC_Ef              154  SFQNYAQQLIQAAQAGNTDTIEIDNGATLKDGTYSLKEKNDSNGYHTTFSMTVKDGKVTESNYDNVNADG  223
Consensus_aa:             ................................................sh.lsK................
Consensus_ss:                                                              eeee
```

```
                                                                               &   *****
Conservation:                                                                  6  5979 7  57
RnfG_Tm              178  -----------------------------------LPKGSPEELKKQGIVKVSDVMTGATITPRAVVTAL  212
RnfG_Tm coords.      178  -----------------------------------LPKGSPEELKKQGIVKVSDV------TPRAVVTAL  212
NqrC_Pd              228  -----------------------------------PGKS----VAGQ---DYVDGISGGTITSKGVDEML  255
NqrC_Pd coords.      228  -----------------------------------PGKS----VAGQ---DYVDGISGGTITSKGVDEML  255
NqrC_Vc              209  ------------------------------------GAP----QGSE---HGVDGLSGATLTSNGVQNTF  235
NqrC_Vc coords.      209  ------------------------------------GAP----QGSE---HGVDGLSGATLTSNGVQNTF  235
NqrC_Vh              213  ------------------------------------GAP----EGSE---HGVDGLSGATLTGNGVQGTF  239
NqrC_Hd              209  -------------------------------------AS----ADKE---HGVDGLSGATLTSKGVDNSF  234
NqrC_So              209  ------------------------------------GVK----EGDI---HGVDAVSGATMTGRGVQRAM  235
NqrC_So coords.      209  ------------------------------------GVK----EGDI---HGVDAVSGATMTGRGVQRAM  235
NqrC_Td              147  --------------------------------------------EVN---ADINAIGGATITSRGVAAIV  169
RnfG_Vc              164  ---------------------------------------------DG---GQFDQFTGATITPRAVVLAV  185
RnfG_Ec              163  ---------------------------------------------DG---GDFDQFTGATITPRAVVNAV  184
NqrC_Ef              224  KSKKDDTEYESKMKDVAGVGPKEYIETLNKEFVKAMGEE----DGSP---AGVEVVTGATHSTHSFINYA  286
Consensus_aa:             ....................................................h-shoGAThTs+tV..hh
Consensus_ss:                                                                 eeeee     hhhhhhhh
```

```
Conservation:                                   5
RnfG_Tm              213  NLMYR--YLEEVSK------------------  224
RnfG_Tm coords.      213  NLMYR--YLEEVSK------------------  224
NqrC_Pd              256  FNSLS--GYVKFLTSQN---------------  270
NqrC_Pd coords.      256  FNSLS--GYVKFLTSQN---------------  270
NqrC_Vc              236  DFWLGDMGFGPFLTKVRDGGLN----------  257
NqrC_Vc coords.      236  DFWLGDMGFGPFLTKVRDGGLN----------  257
NqrC_Vh              240  DFWLGDMGFGPFLAKVRDGGLN----------  261
NqrC_Hd              235  KYWFGTNGFGPYLAKFKATAGAN---------  257
NqrC_So              236  EFWFGVEGFQTFFNQLKASADQGELGGAK---  264
NqrC_So coords.      226  EFWFGVEGFQTFFNQLKAS-------------  253
NqrC_Td              170  KDASV--TAIEYMAKNNLEEGK----------  189
RnfG_Vc              186  KKAVE--YVNQHQQQLHNQPNPCEGQ------  209
RnfG_Ec              185  KRAGL--YAQTLPAQLSQLPACGE--------  206
NqrC_Ef              287  QQLVN--AAEKGDT-------TEIVVDNIVTK  309
Consensus_aa:             p.hh....h..h..p.p...............
Consensus_ss:             hhhhh  hhhhhhhhhh
```

## Figure S2.

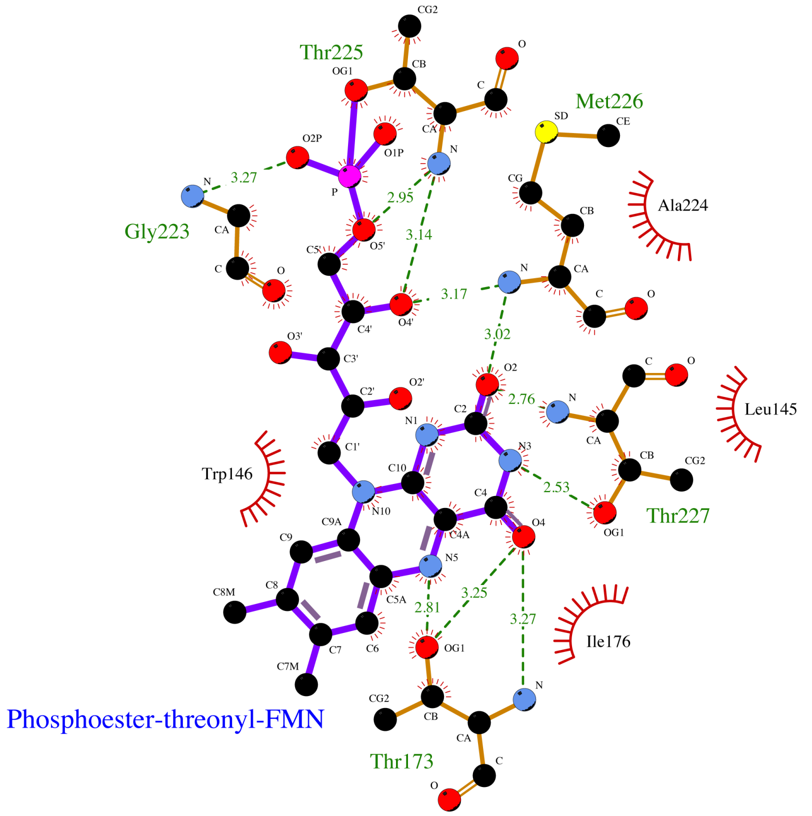
Thr225
Met226
Ala224
Gly223
Leu145
Trp146
Thr227
Ile176
Phosphoester-threonyl-FMN
Thr173
3.27
2.95
3.14
3.17
3.02
2.76
2.53
2.81
3.25
3.27
